# Supplementary material for: Non-Targeted LC-MS/MS Assay for Screening Over 100 Lipid Mediators from ARA, EPA, and DHA in Biological Samples Based on Mass Spectral Fragmentations
Source: Molecules. 2019 Jun 19;24(12):2276. doi: 10.3390/molecules24122276 (PMC6630234; doi:10.3390/molecules24122276)

Supplementary Material S1: Common and specific transitions suggested for hydroxy-derivates from ARA, EPA and DHA. Product ion spectrum at *m/z* 319 for HETEs, *m/z* 317 for HEPEs, and *m/z* 343 for HDoHEs were obtained from database of *LIPID MAPS* at CID 30V. No spectra was found for 19-HETE.

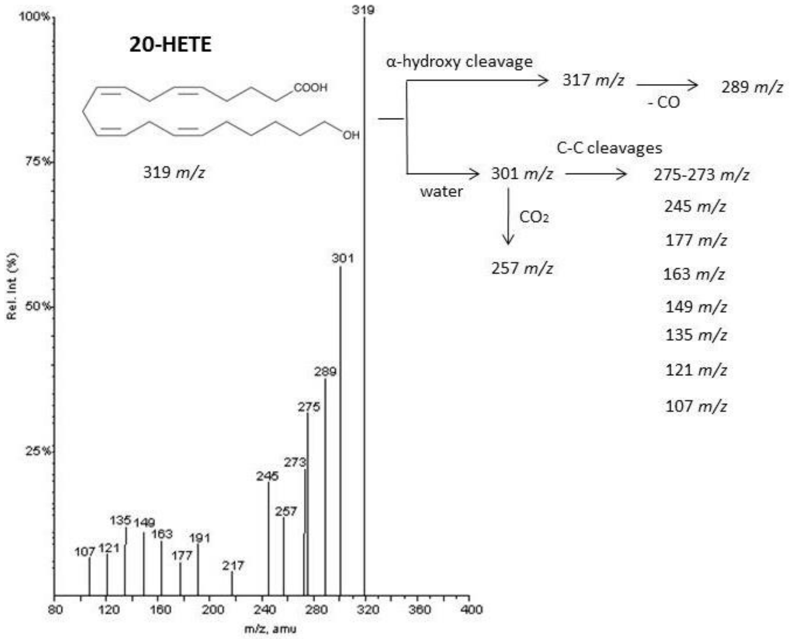

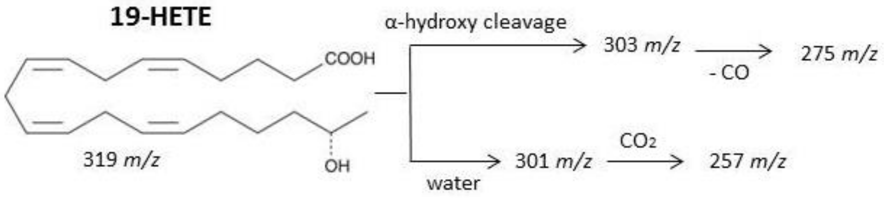

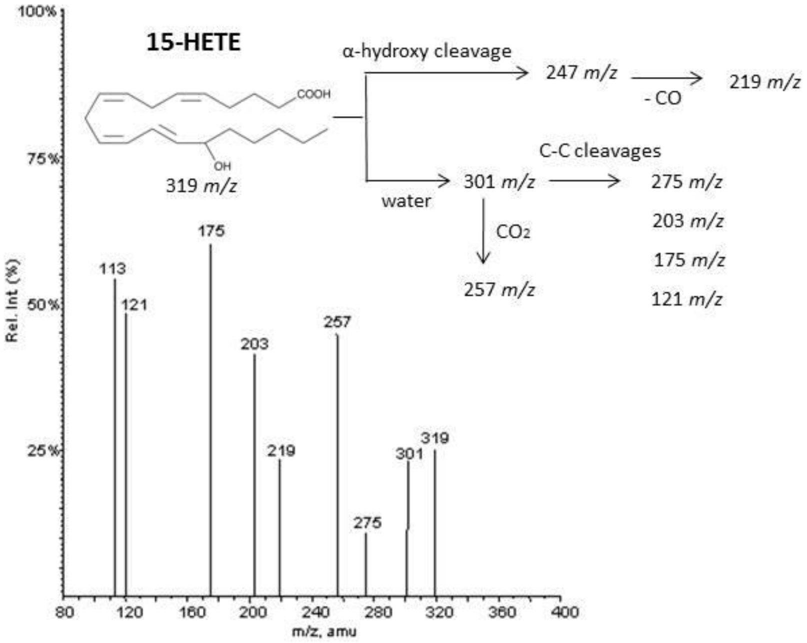

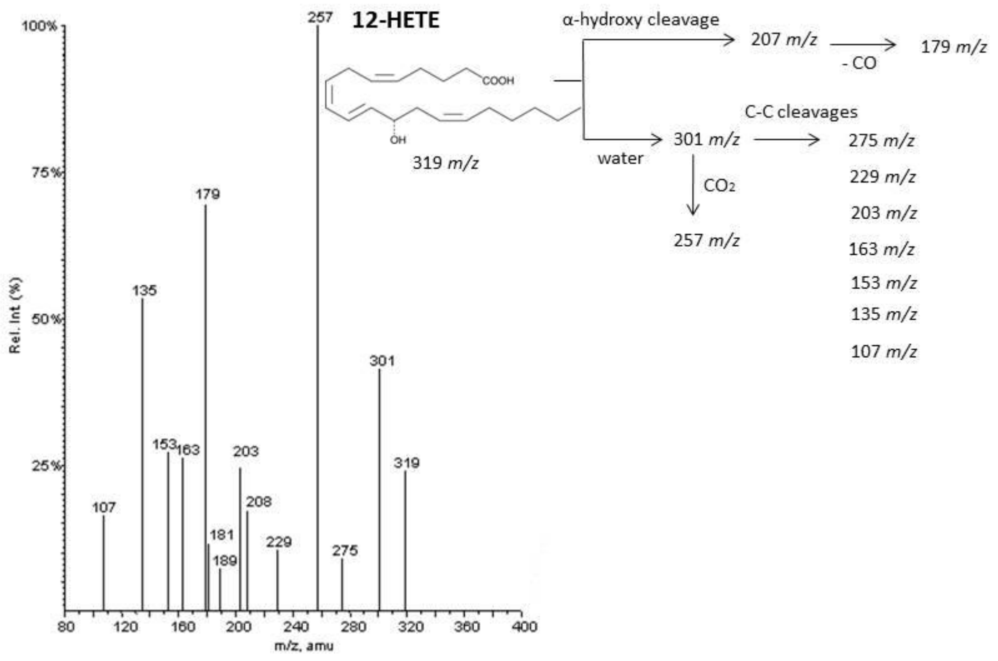
12-HETE
α-hydroxy cleavage
207 m/z
- CO
179 m/z
COOH
OH
319 m/z
water
301 m/z
C-C cleavages
275 m/z
229 m/z
203 m/z
163 m/z
153 m/z
135 m/z
107 m/z
CO2
257 m/z
Rel. Int (%)
m/z, amu

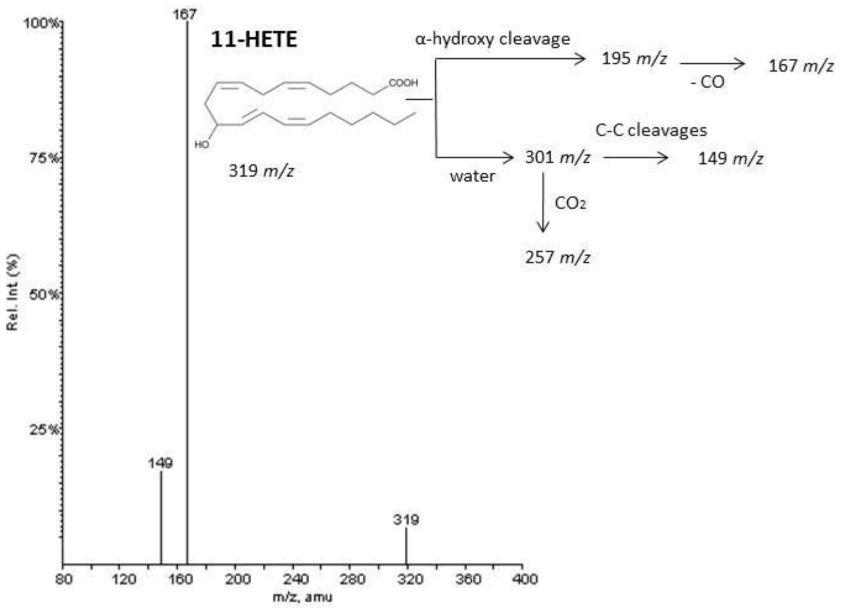
11-HETE
α-hydroxy cleavage
195 m/z
- CO
167 m/z
COOH
HO
319 m/z
water
301 m/z
C-C cleavages
149 m/z
CO2
257 m/z
Rel. Int (%)
m/z, amu

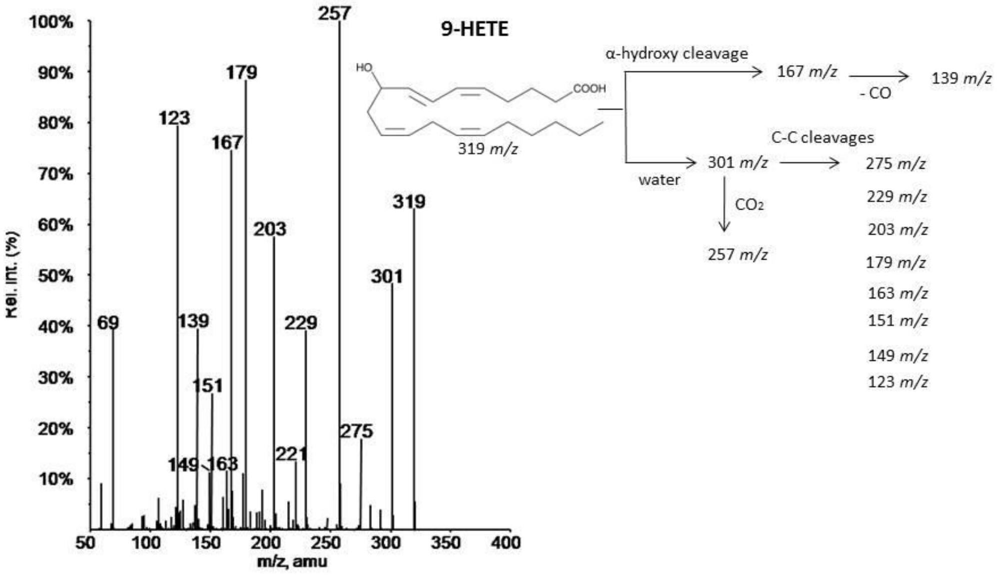
9-HETE
α-hydroxy cleavage
167 m/z
- CO
139 m/z
HO
COOH
319 m/z
water
301 m/z
C-C cleavages
275 m/z
229 m/z
203 m/z
179 m/z
163 m/z
151 m/z
149 m/z
123 m/z
CO2
257 m/z
Rel. Int. (%)
m/z, amu

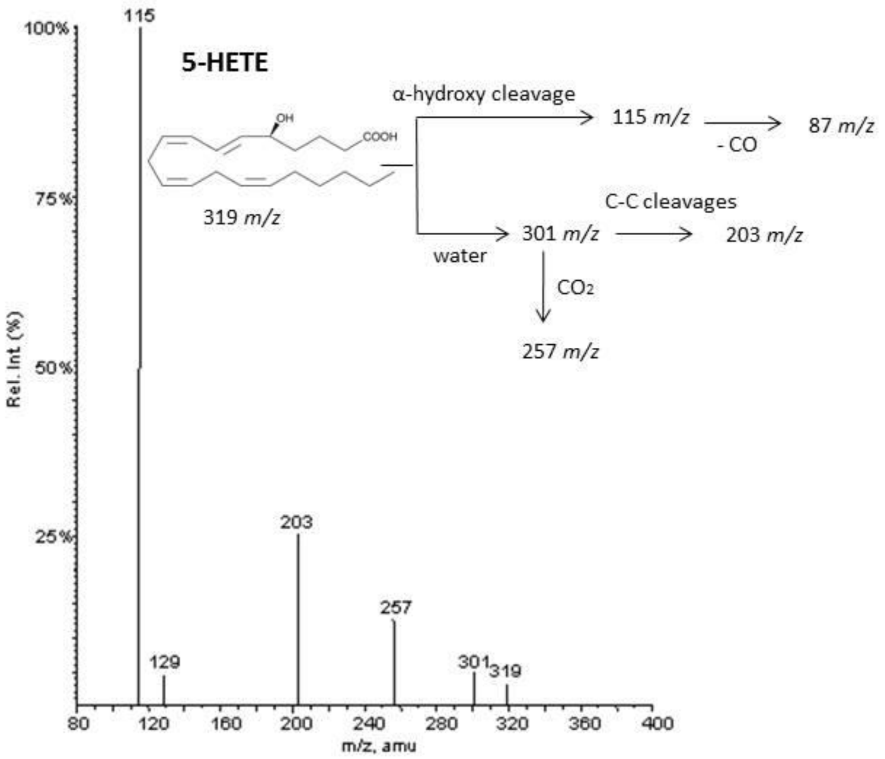
5-HETE
α-hydroxy cleavage
115 m/z
- CO
87 m/z
319 m/z
C-C cleavages
water
301 m/z
203 m/z
CO2
257 m/z
Rel. Int (%)
m/z, amu
115
203
257
129
301
319

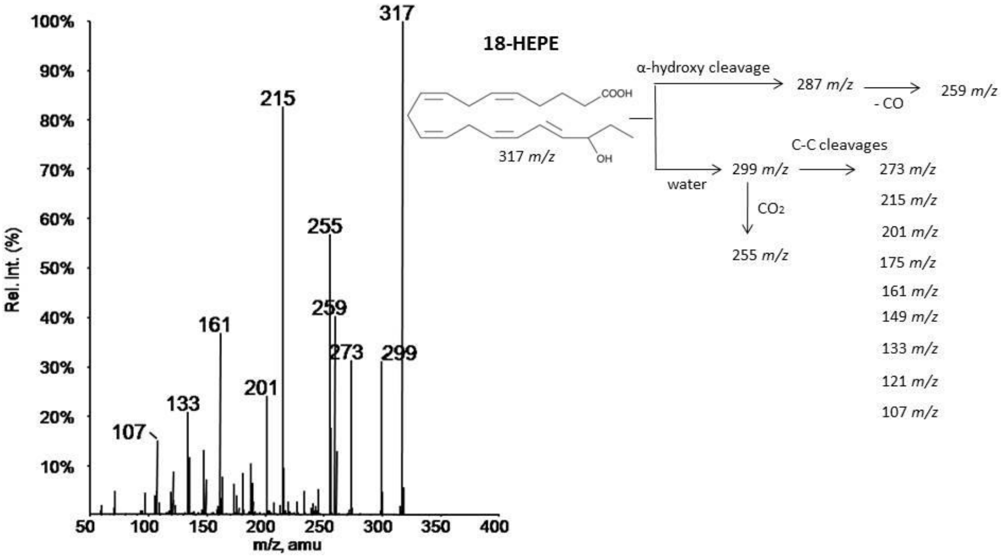
18-HEPE
α-hydroxy cleavage
287 m/z
- CO
259 m/z
317 m/z
C-C cleavages
water
299 m/z
CO2
255 m/z
273 m/z
215 m/z
201 m/z
175 m/z
161 m/z
149 m/z
133 m/z
121 m/z
107 m/z
Rel. Int. (%)
m/z, amu

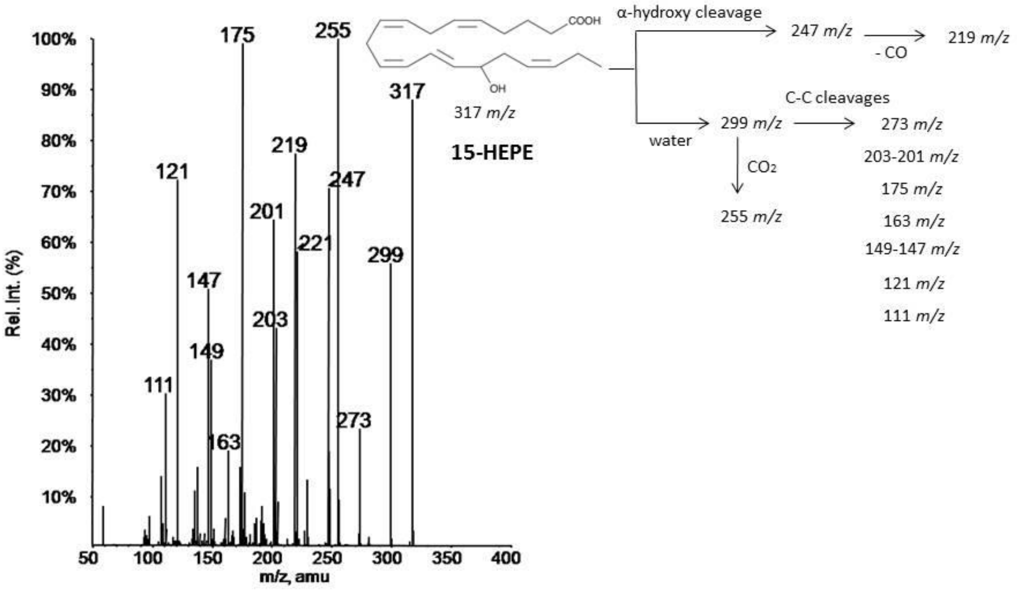
15-HEPE
α-hydroxy cleavage
247 m/z
- CO
219 m/z
317 m/z
C-C cleavages
water
299 m/z
CO2
255 m/z
273 m/z
203-201 m/z
175 m/z
163 m/z
149-147 m/z
121 m/z
111 m/z
Rel. Int. (%)
m/z, amu

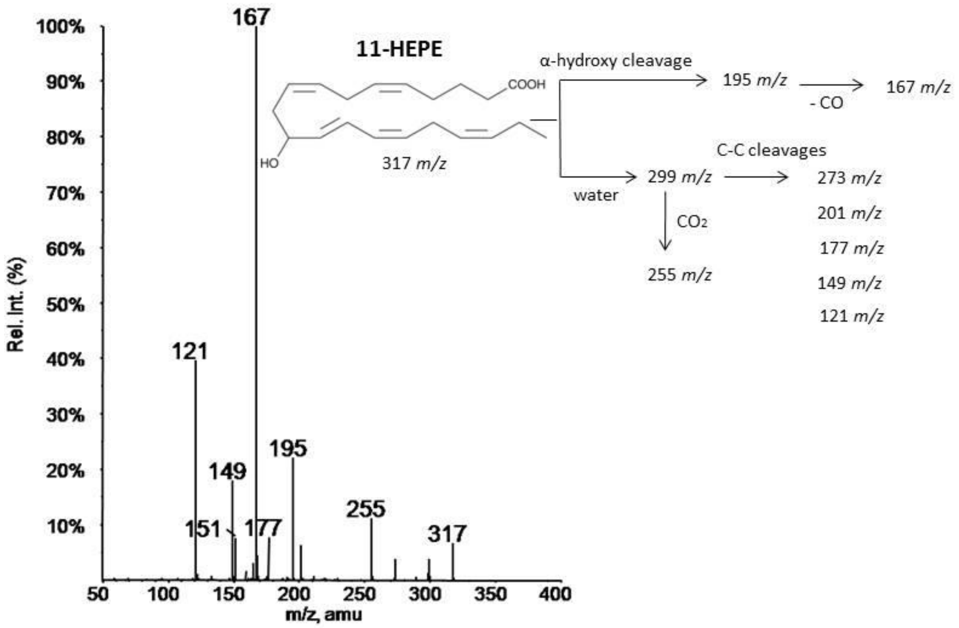
11-HEPE
COOH
HO
317 m/z
α-hydroxy cleavage
195 m/z
- CO
167 m/z
water
299 m/z
C-C cleavages
273 m/z
201 m/z
177 m/z
149 m/z
121 m/z
CO2
255 m/z
Rel. Int. (%)
m/z, amu
167
121
149
151
177
195
255
317

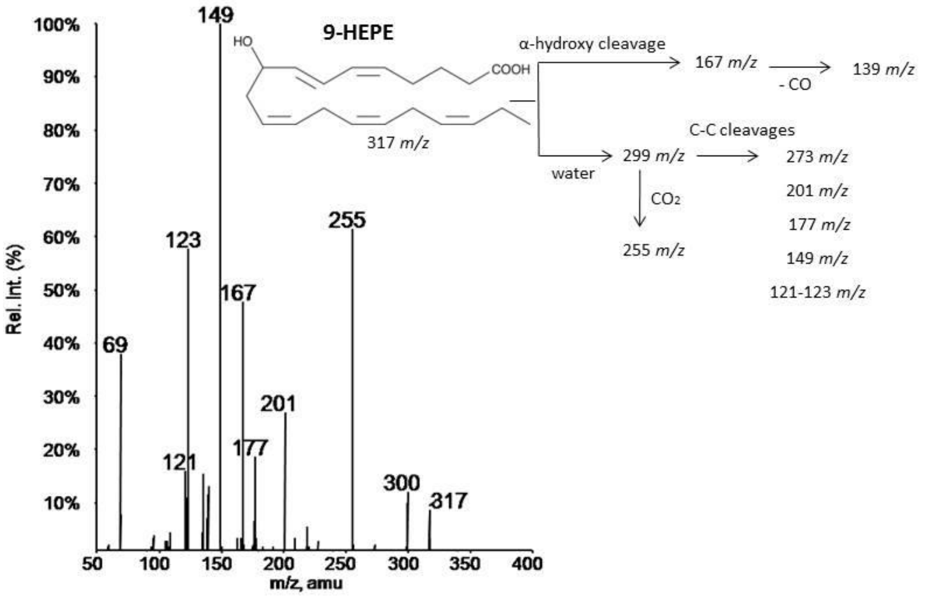
9-HEPE
HO
COOH
317 m/z
α-hydroxy cleavage
167 m/z
- CO
139 m/z
water
299 m/z
C-C cleavages
273 m/z
201 m/z
177 m/z
149 m/z
121-123 m/z
CO2
255 m/z
Rel. Int. (%)
m/z, amu
149
123
255
167
69
201
121
177
300
317

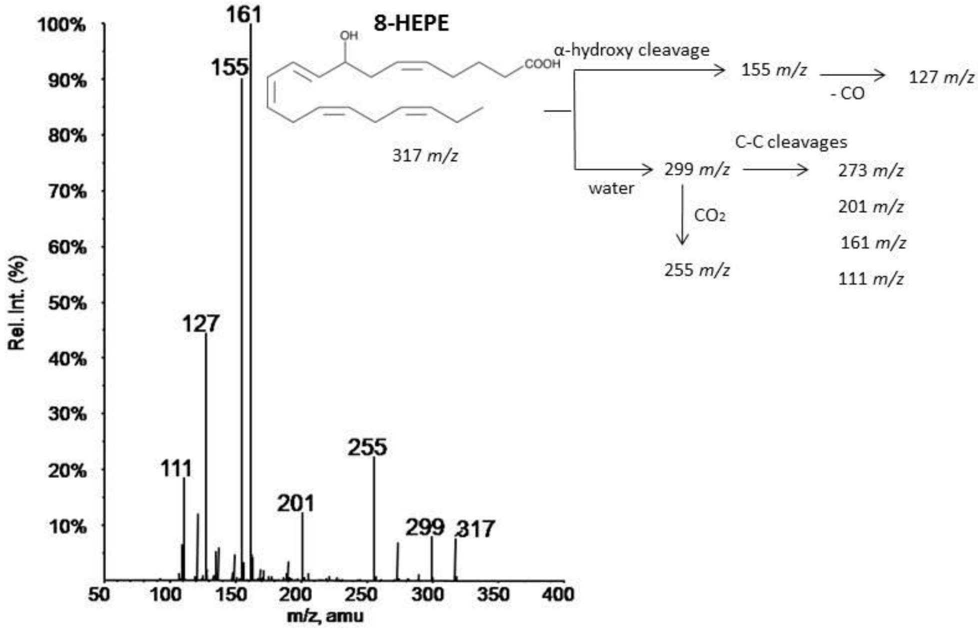
8-HEPE
OH
COOH
317 m/z
α-hydroxy cleavage
155 m/z
- CO
127 m/z
water
299 m/z
C-C cleavages
273 m/z
201 m/z
161 m/z
111 m/z
CO2
255 m/z
Rel. Int. (%)
m/z, amu
161
155
127
111
201
255
299
317

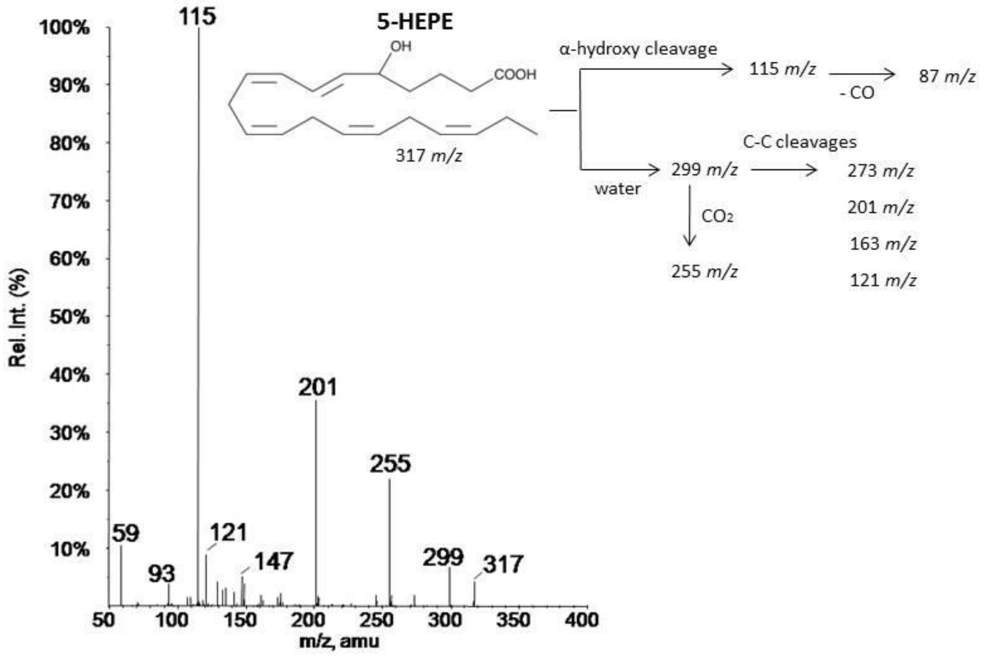

5-HEPE
α-hydroxy cleavage
115 m/z
- CO
87 m/z
317 m/z
C-C cleavages
water
299 m/z
273 m/z
201 m/z
163 m/z
121 m/z
CO2
255 m/z
Rel. Int. (%)
m/z, amu
115
201
255
59
93
121
147
299
317

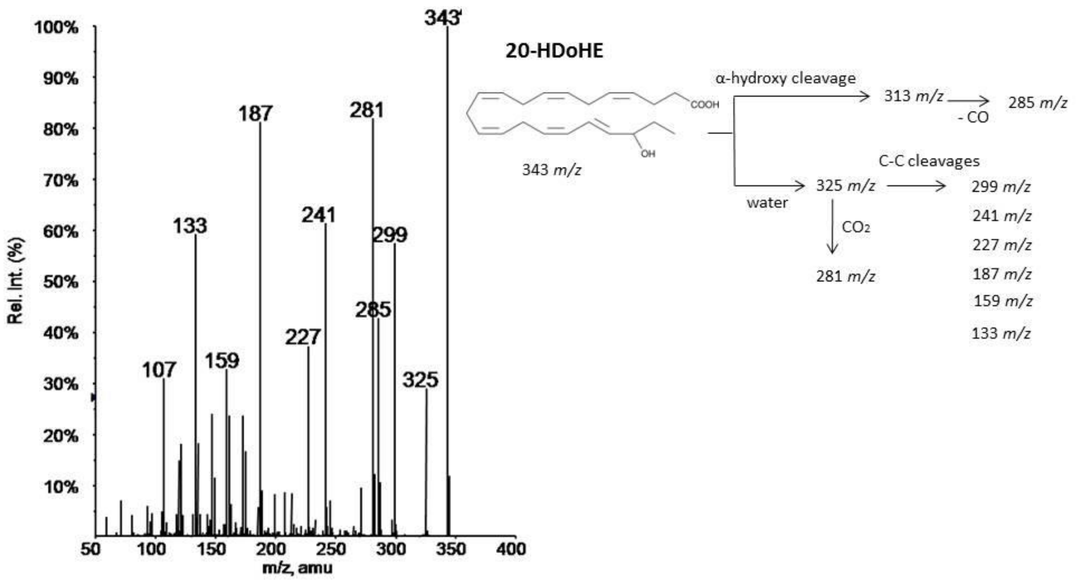

20-HDoHE
α-hydroxy cleavage
313 m/z
- CO
285 m/z
343 m/z
C-C cleavages
water
325 m/z
299 m/z
241 m/z
227 m/z
187 m/z
159 m/z
133 m/z
CO2
281 m/z
Rel. Int. (%)
m/z, amu
343
187
281
133
241
299
285
227
107
159
325

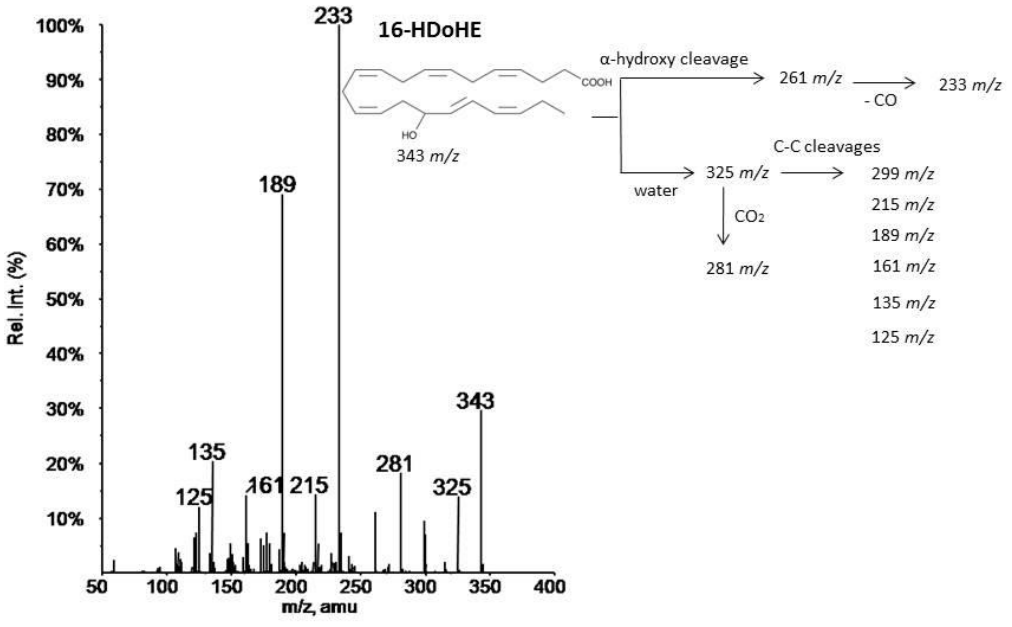

16-HDoHE
α-hydroxy cleavage
261 m/z
- CO
233 m/z
343 m/z
C-C cleavages
water
325 m/z
299 m/z
215 m/z
189 m/z
161 m/z
135 m/z
125 m/z
CO2
281 m/z
Rel. Int. (%)
m/z, amu
233
189
343
135
125
161
215
281
325

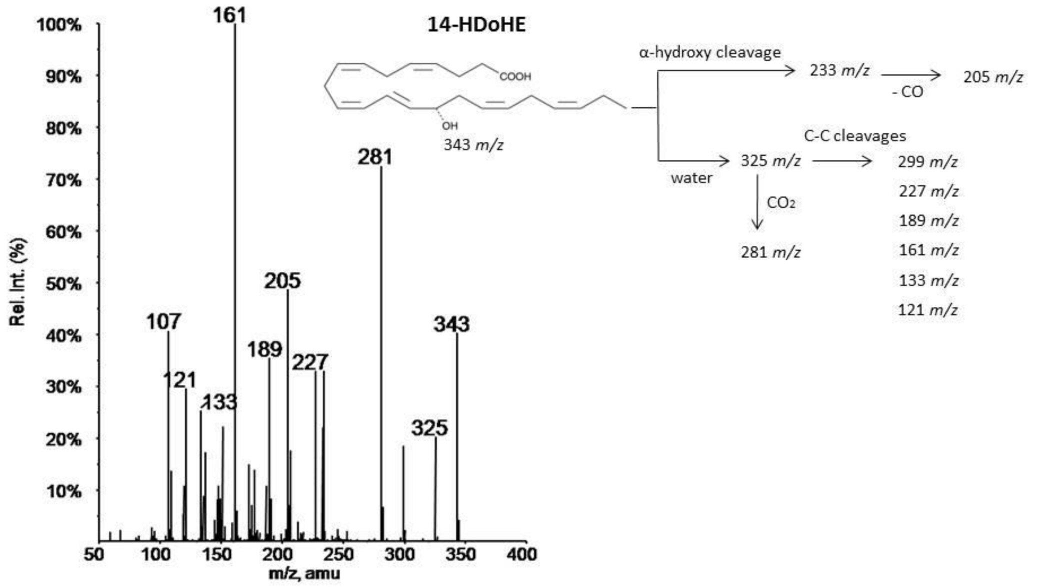
14-HDoHE
COOH
OH
343 m/z
α-hydroxy cleavage
233 m/z
- CO
205 m/z
C-C cleavages
water
325 m/z
CO2
281 m/z
299 m/z
227 m/z
189 m/z
161 m/z
133 m/z
121 m/z
Rel. Int. (%)
m/z, amu
161
281
205
107
189
227
343
121
133
325

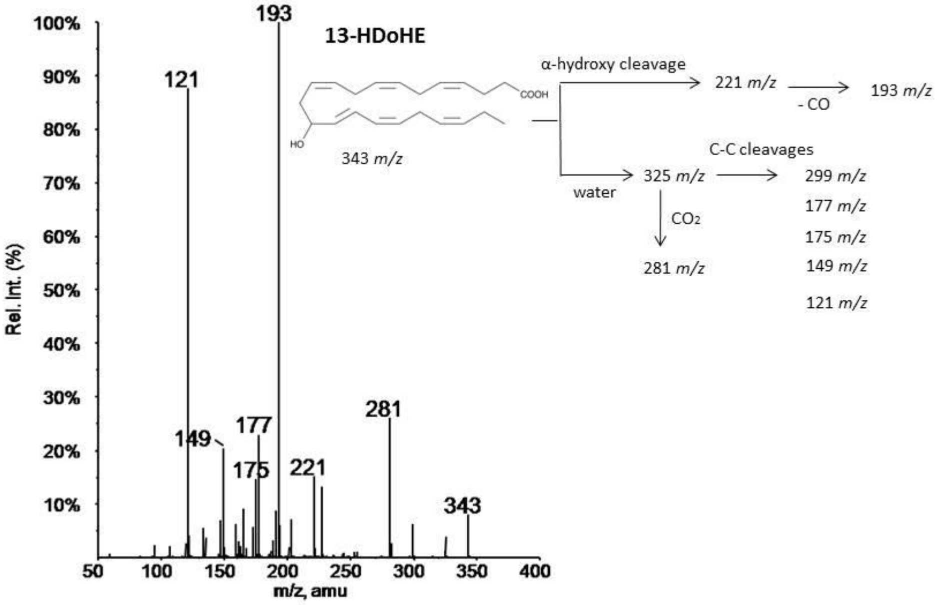
13-HDoHE
COOH
HO
343 m/z
α-hydroxy cleavage
221 m/z
- CO
193 m/z
C-C cleavages
water
325 m/z
CO2
281 m/z
299 m/z
177 m/z
175 m/z
149 m/z
121 m/z
Rel. Int. (%)
m/z, amu
193
121
281
149
177
175
221
343

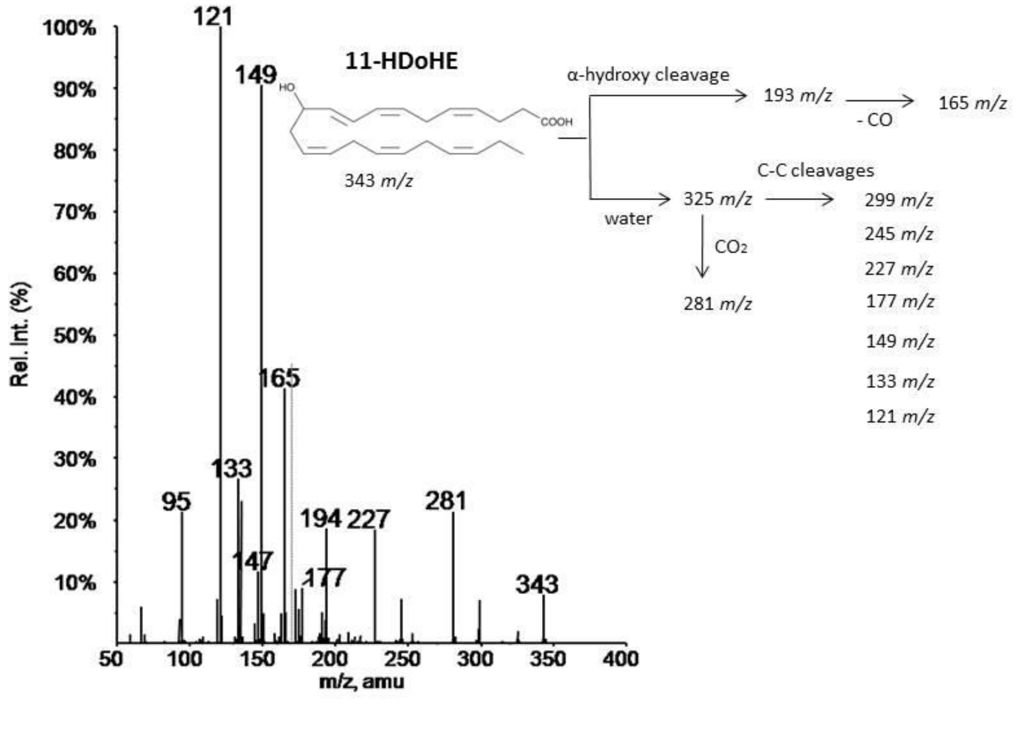
11-HDoHE
HO
COOH
343 m/z
α-hydroxy cleavage
193 m/z
- CO
165 m/z
C-C cleavages
water
325 m/z
CO2
281 m/z
299 m/z
245 m/z
227 m/z
177 m/z
149 m/z
133 m/z
121 m/z
Rel. Int. (%)
m/z, amu
121
149
165
133
95
194
227
281
147
177
343

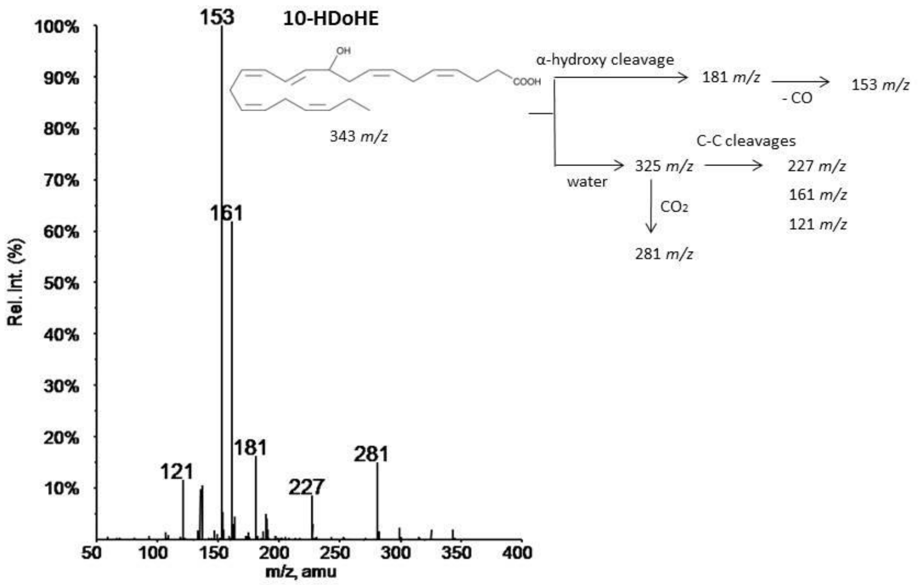
10-HDoHE
343 m/z
α-hydroxy cleavage
181 m/z
- CO
153 m/z
water
325 m/z
C-C cleavages
227 m/z
161 m/z
121 m/z
CO2
281 m/z
Rel. Int. (%)
m/z, amu
153
161
121
181
227
281

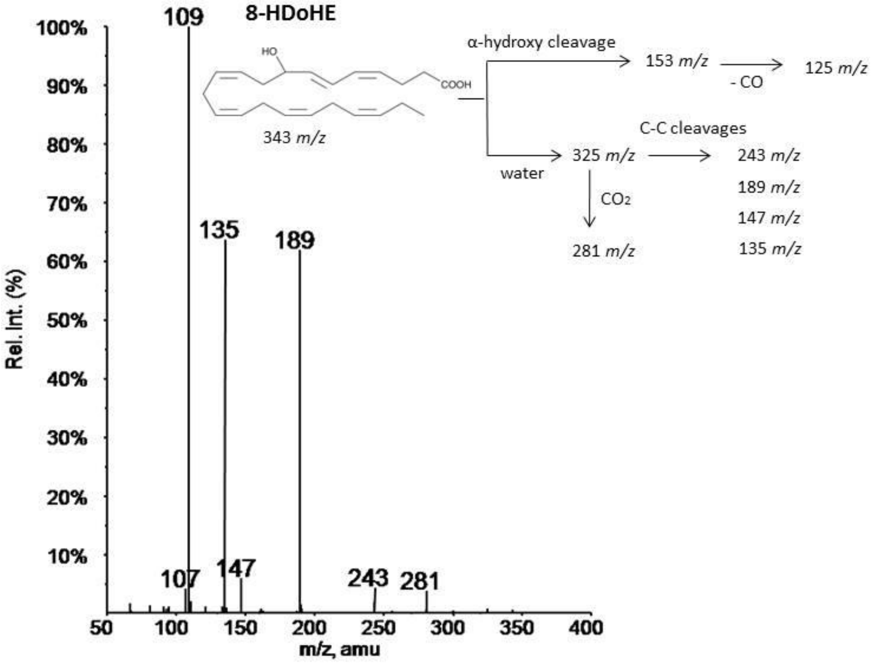
8-HDoHE
343 m/z
α-hydroxy cleavage
153 m/z
- CO
125 m/z
water
325 m/z
C-C cleavages
243 m/z
189 m/z
147 m/z
135 m/z
CO2
281 m/z
Rel. Int. (%)
m/z, amu
109
135
189
107
147
243
281

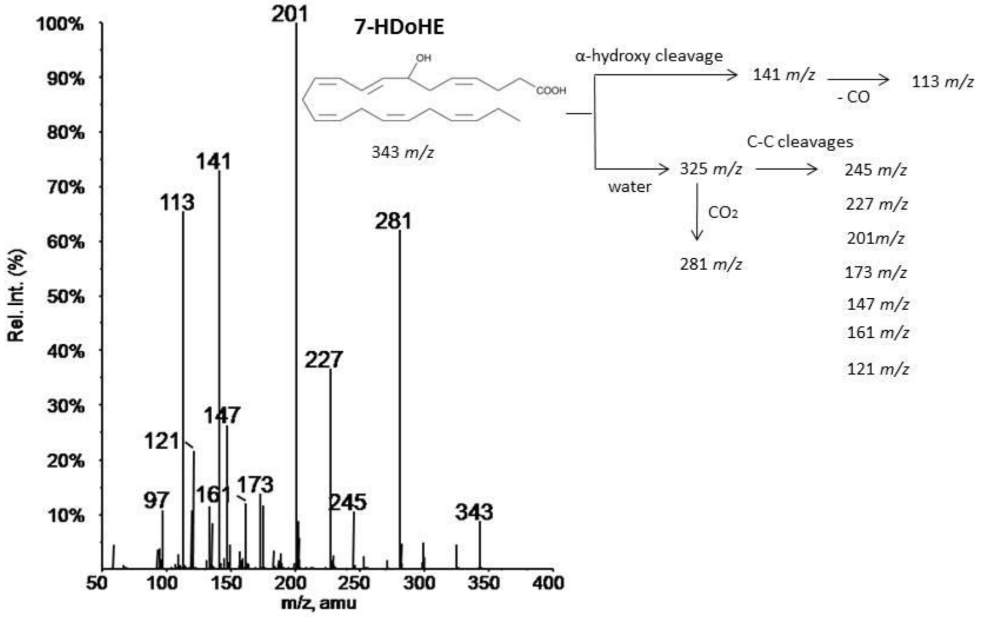
7-HDoHE
343 m/z
α-hydroxy cleavage
141 m/z
- CO
113 m/z
water
325 m/z
C-C cleavages
245 m/z
227 m/z
201m/z
173 m/z
147 m/z
161 m/z
121 m/z
CO2
281 m/z
Rel. Int. (%)
m/z, amu
201
141
113
281
227
121
147
97
161
173
245
343

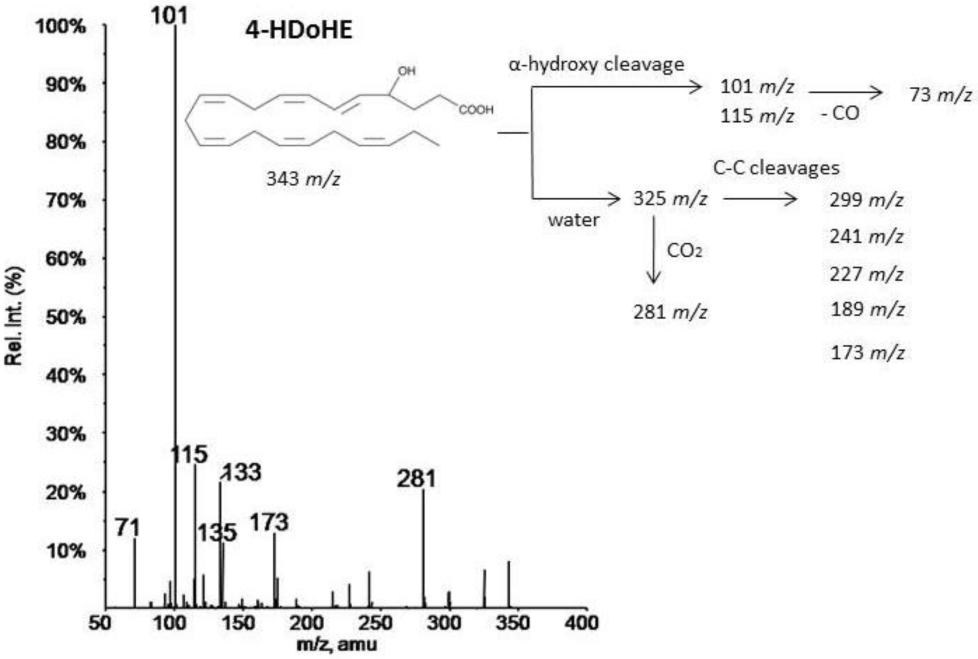
4-HDoHE
100%
90%
80%
70%
60%
50%
40%
30%
20%
10%
Rel. Int. (%)
101
115
133
135
173
281
71
50
100
150
200
250
300
350
400
m/z, amu
OH
COOH
343 m/z
α-hydroxy cleavage
101 m/z
115 m/z
- CO
73 m/z
C-C cleavages
water
325 m/z
299 m/z
241 m/z
227 m/z
189 m/z
173 m/z
CO2
281 m/z

Supplementary Material S2: Common and specific transitions suggested for 12 and 15 hydroperoxy-derivates of ARA and EPA. Product ion spectrum at *m/z* 335 for HpETEs, and *m/z* 333 for HpEPEs were obtained from database of *LIPID MAPS* at CID 30V.

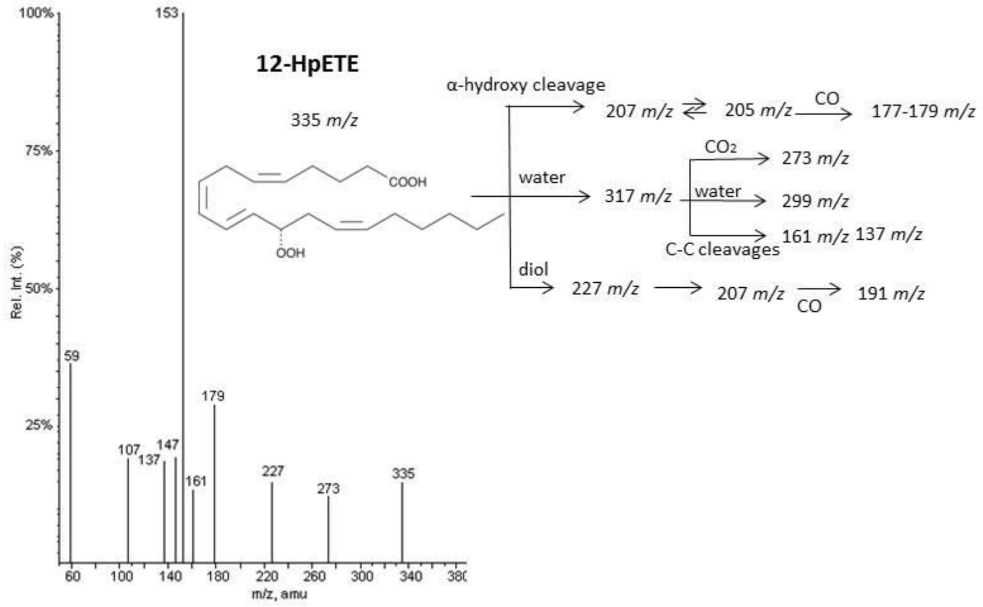

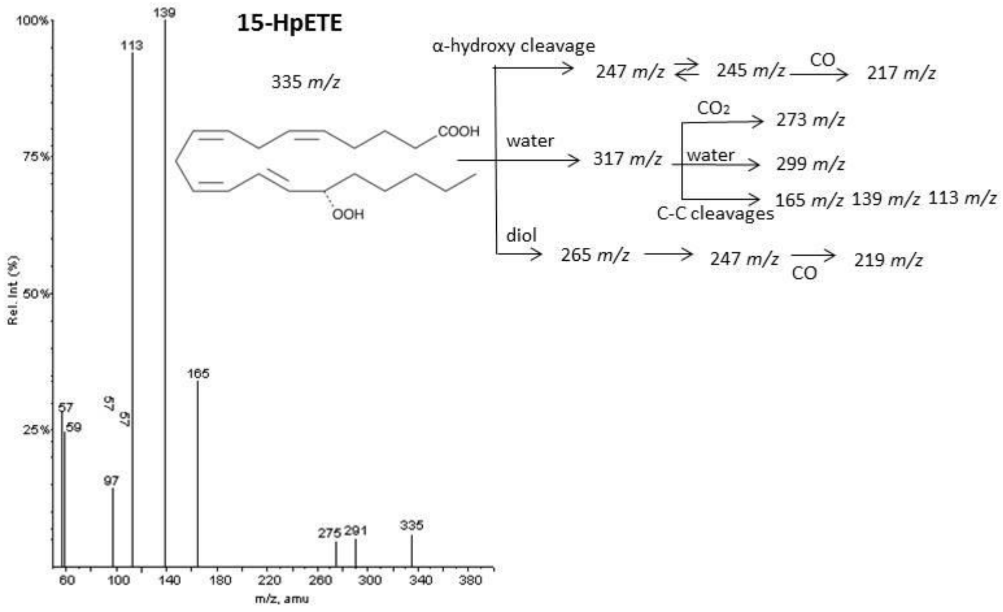

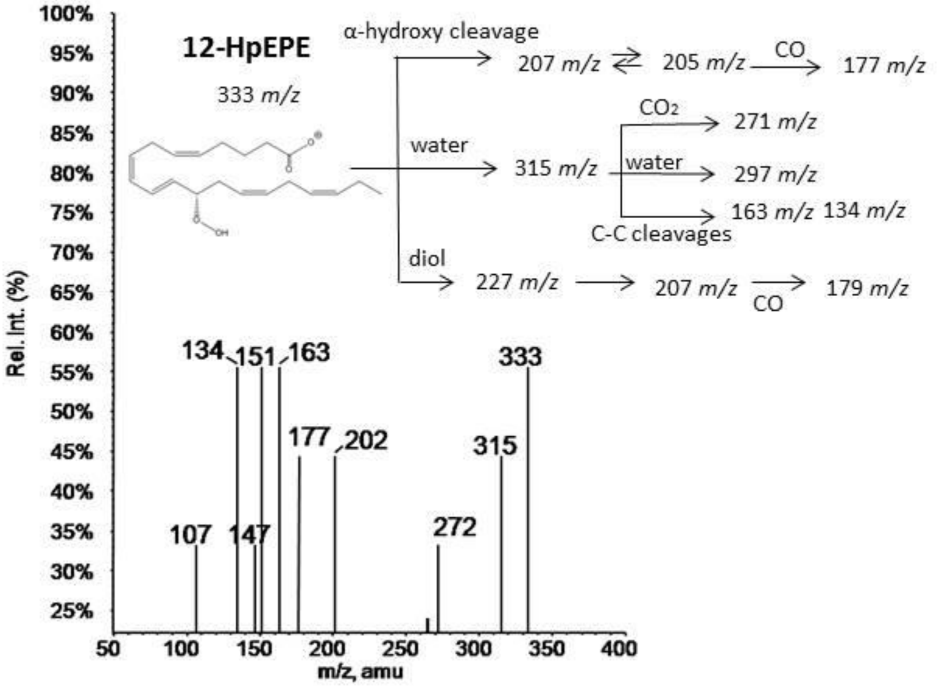
12-HpEPE
333 m/z
α-hydroxy cleavage
207 m/z ⇄ 205 m/z → CO → 177 m/z
water
315 m/z
CO2 → 271 m/z
water → 297 m/z
163 m/z 134 m/z
C-C cleavages
diol
227 m/z → 207 m/z → CO → 179 m/z
Rel. Int. (%)
m/z, amu
134 151 163 333 177 202 315 107 147 272

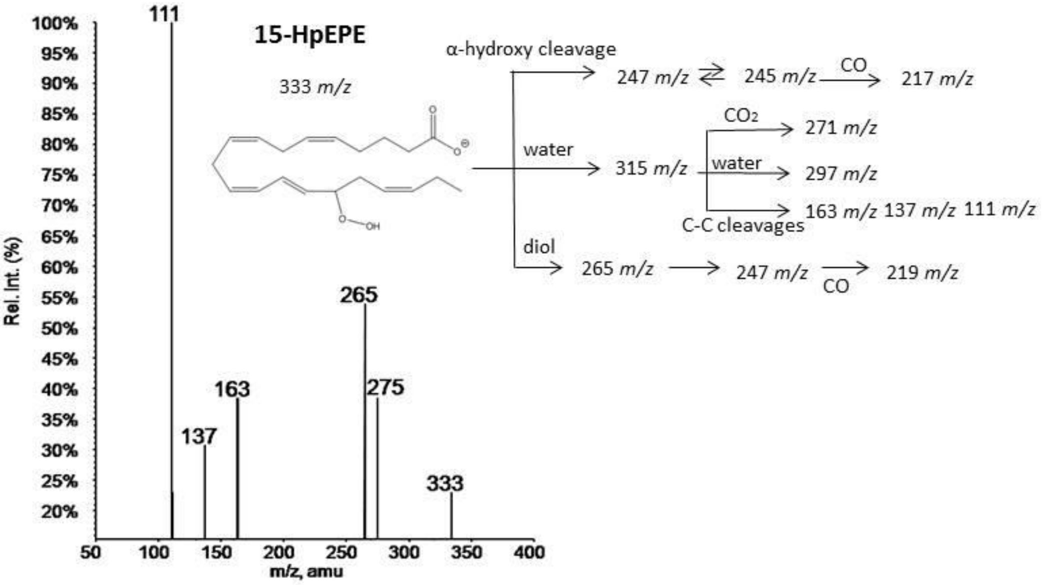
15-HpEPE
333 m/z
α-hydroxy cleavage
247 m/z ⇄ 245 m/z → CO → 217 m/z
water
315 m/z
CO2 → 271 m/z
water → 297 m/z
163 m/z 137 m/z 111 m/z
C-C cleavages
diol
265 m/z → 247 m/z → CO → 219 m/z
Rel. Int. (%)
m/z, amu
111 265 163 275 137 333

Supplementary Materials S3: Fragmentation pattern suggested for prostaglandins derived from ARA and EPA. Product ion spectrum at *m/z* 331, *m/z* 333, *m/z* 351, and *m/z* 367 were obtained from database of *LIPID MAPS* at CID 30V.

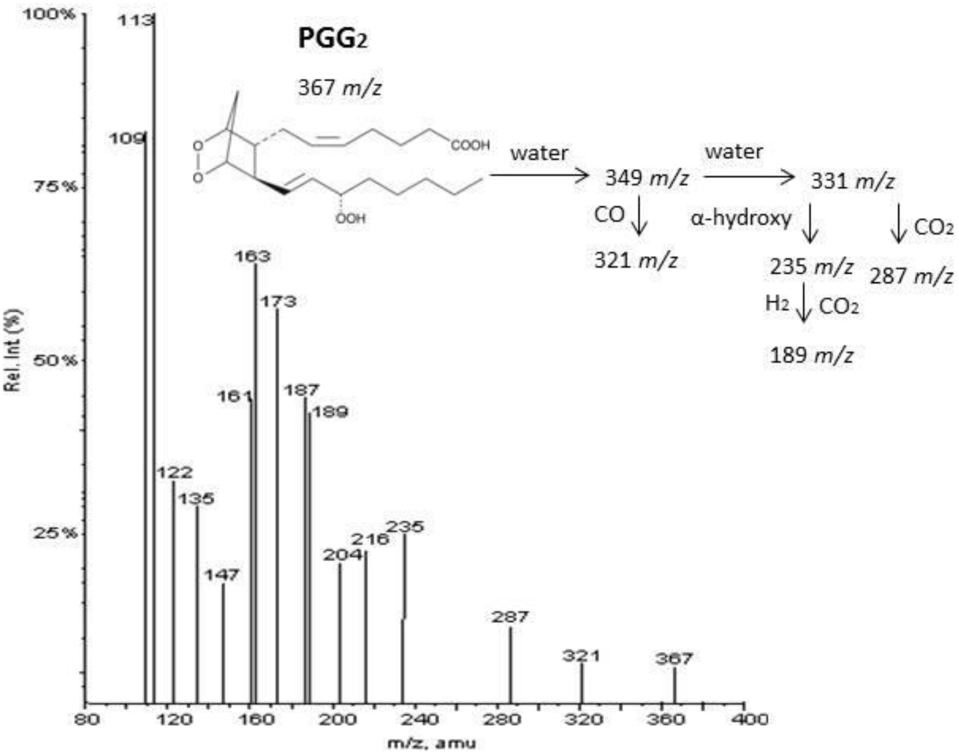

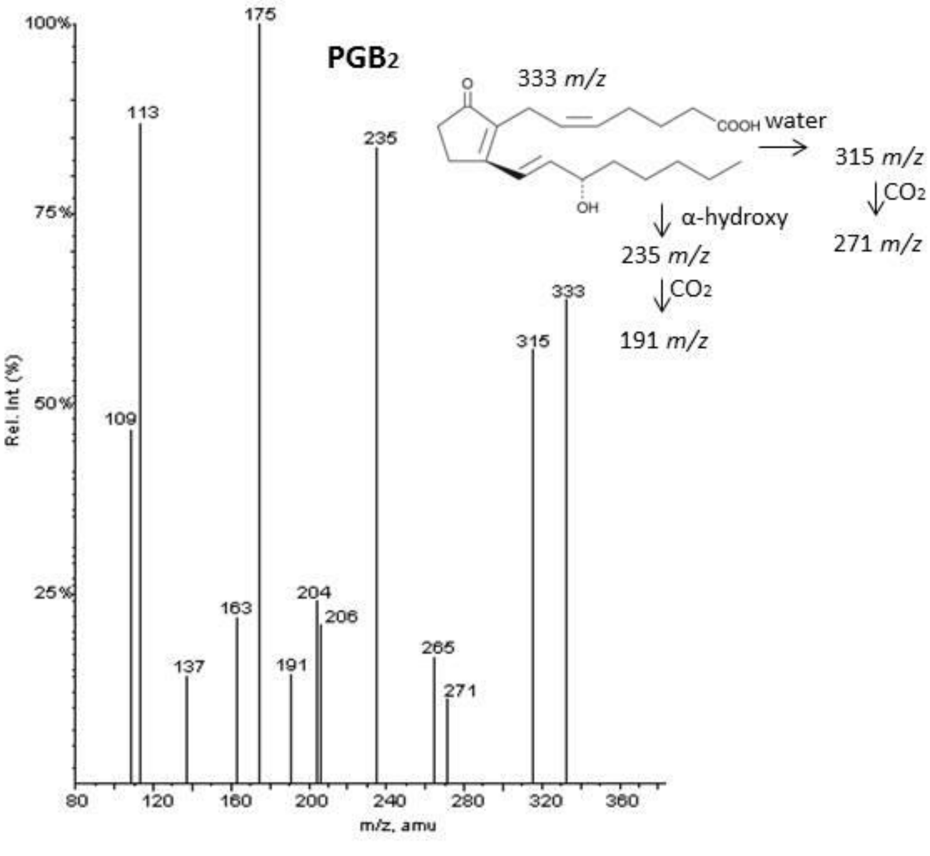

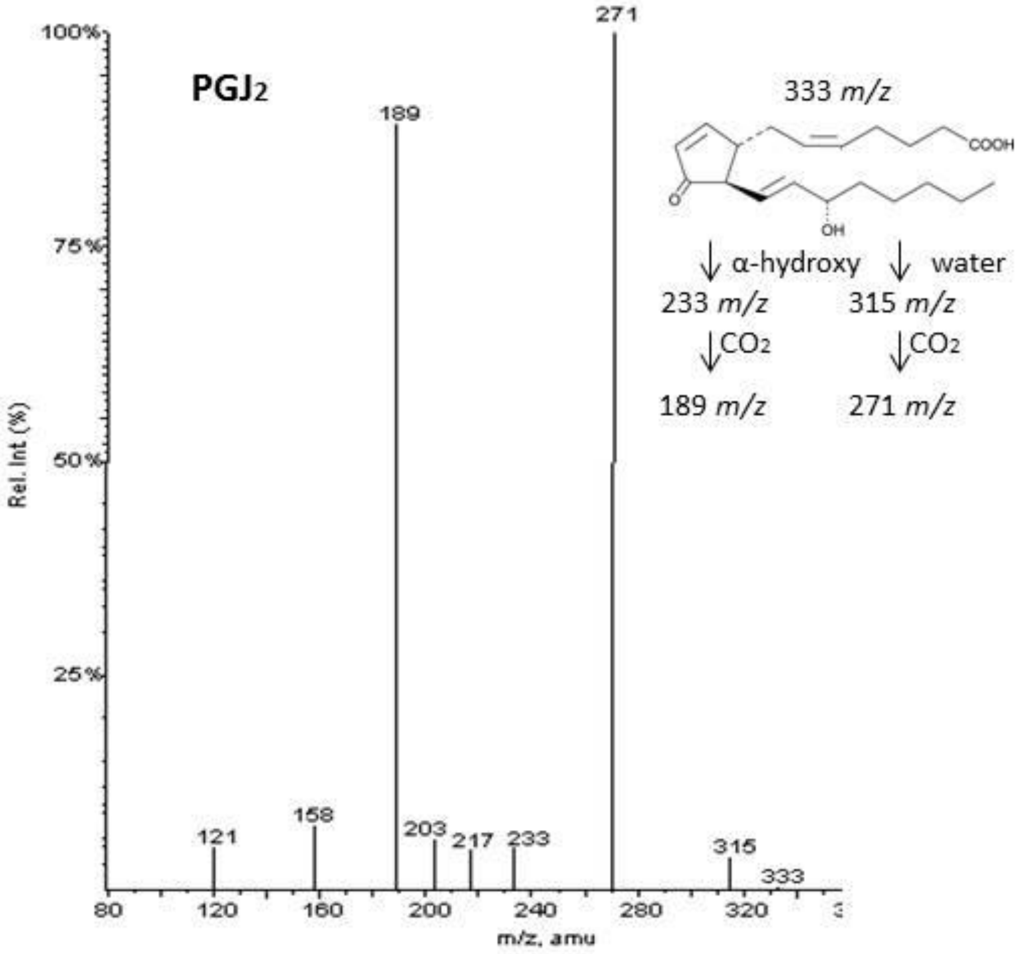
PGJ2
333 m/z
COOH
O
OH
α-hydroxy
water
233 m/z
315 m/z
CO2
CO2
189 m/z
271 m/z
100%
75%
50%
25%
Rel. Int. (%)
271
189
121
158
203
217
233
315
333
80
120
160
200
240
280
320
m/z, amu

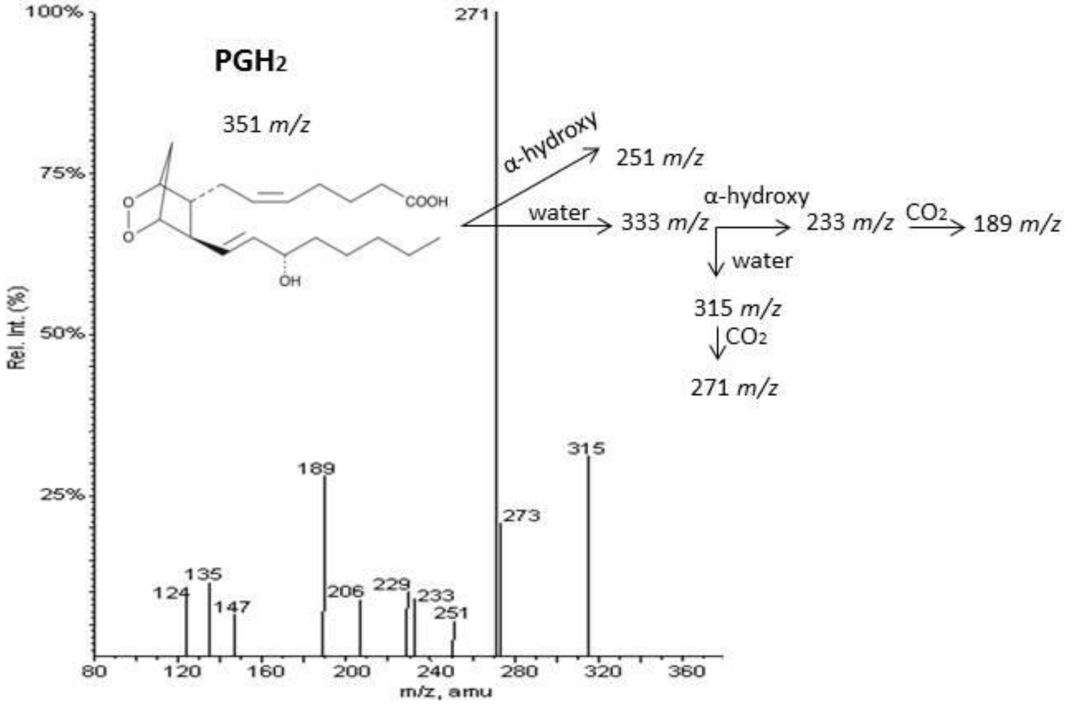
PGH2
351 m/z
COOH
OH
α-hydroxy
251 m/z
water
333 m/z
α-hydroxy
233 m/z
CO2
189 m/z
water
315 m/z
CO2
271 m/z
100%
75%
50%
25%
Rel. Int. (%)
271
315
189
273
124
135
147
206
229
233
251
80
120
160
200
240
280
320
360
m/z, amu

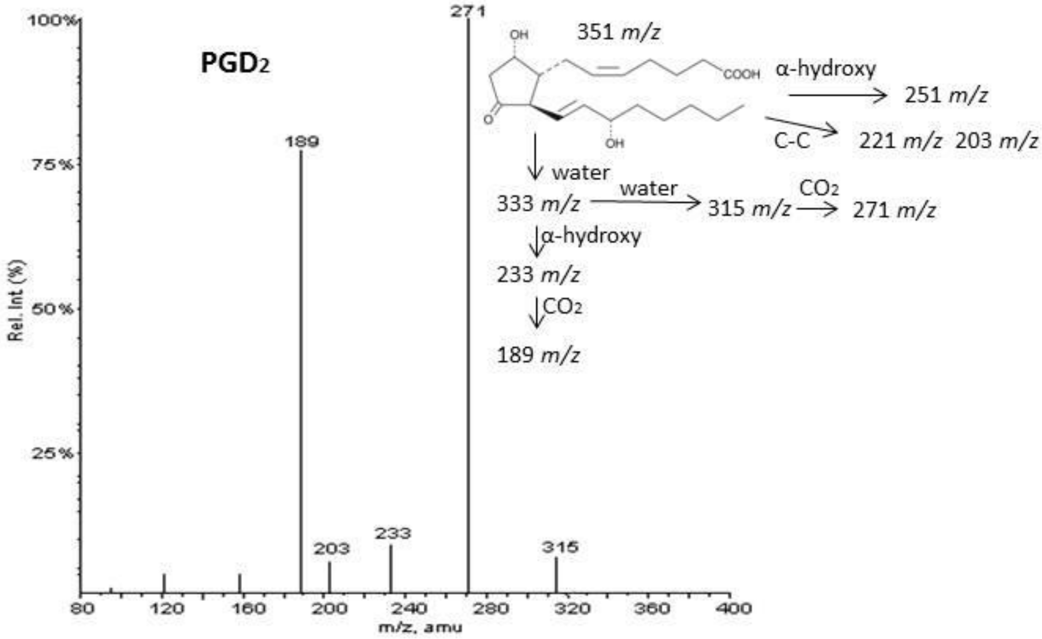
PGD2
351 m/z
α-hydroxy
251 m/z
C-C
221 m/z 203 m/z
water
333 m/z
315 m/z
CO2
271 m/z
233 m/z
189 m/z
Rel. Int (%)
m/z, amu

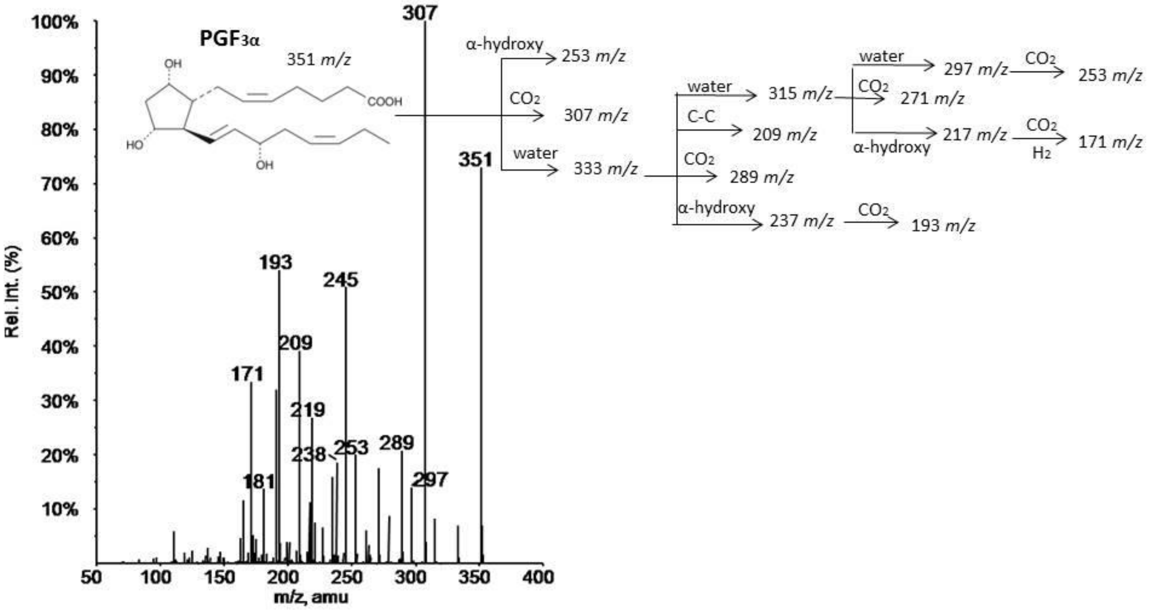
PGF3α
351 m/z
α-hydroxy
253 m/z
CO2
307 m/z
water
333 m/z
315 m/z
297 m/z
271 m/z
217 m/z
171 m/z
H2
C-C
209 m/z
289 m/z
237 m/z
193 m/z
Rel. Int. (%)
m/z, amu

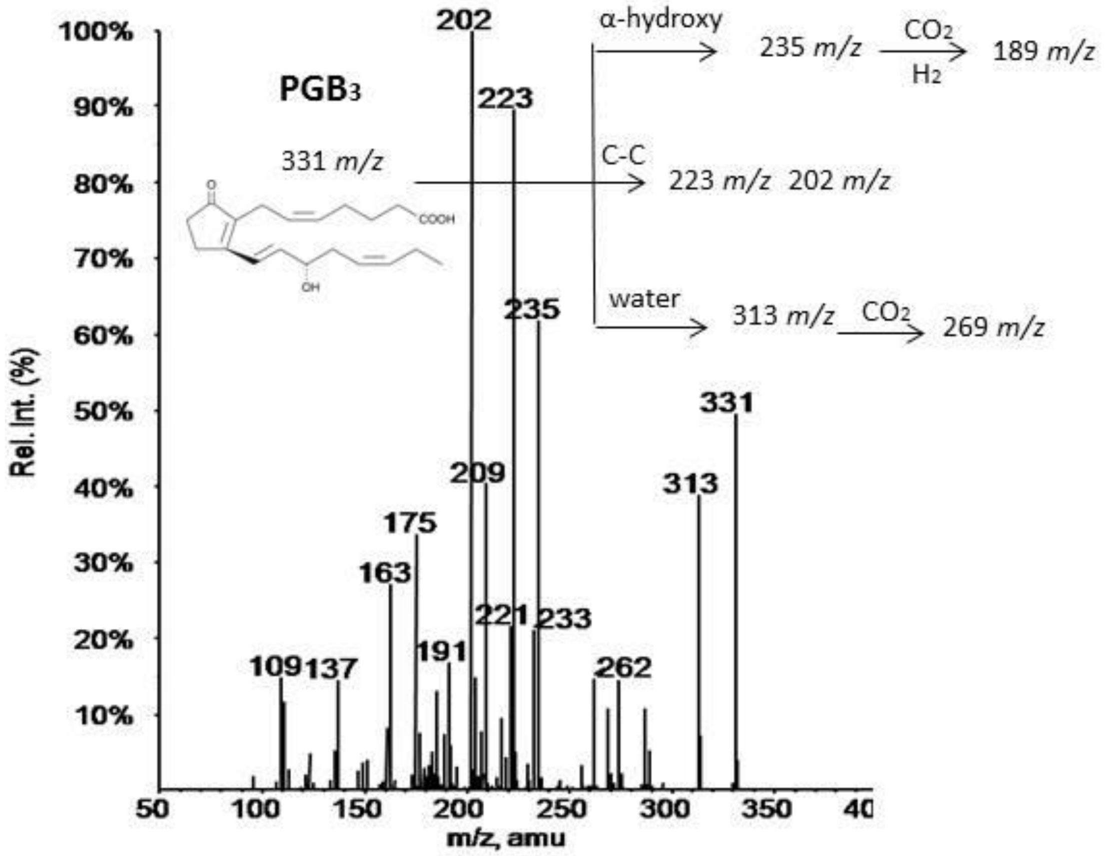
PGB3
331 m/z
α-hydroxy
235 m/z
CO2
H2
189 m/z
C-C
223 m/z 202 m/z
water
313 m/z
CO2
269 m/z
Rel. Int. (%)
m/z, amu
202
223
235
331
209
313
175
163
221
233
191
109
137
262

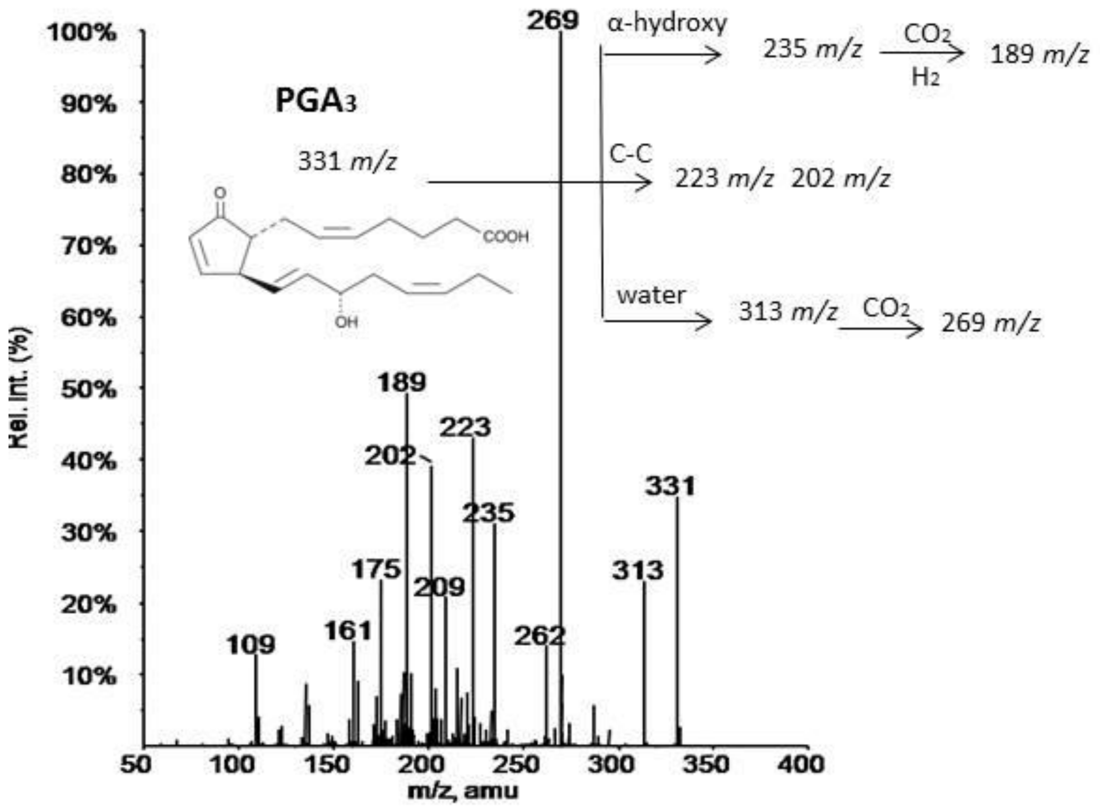
PGA3
331 m/z
α-hydroxy
235 m/z
CO2
H2
189 m/z
C-C
223 m/z 202 m/z
water
313 m/z
CO2
269 m/z
Rel. Int. (%)
m/z, amu
269
189
223
202
331
235
175
209
313
161
109
262

Supplementary Materials S4: Possible fragments for identification of RvD_2, RvD_3, RvD_4, RvE_2, and MaR-1.

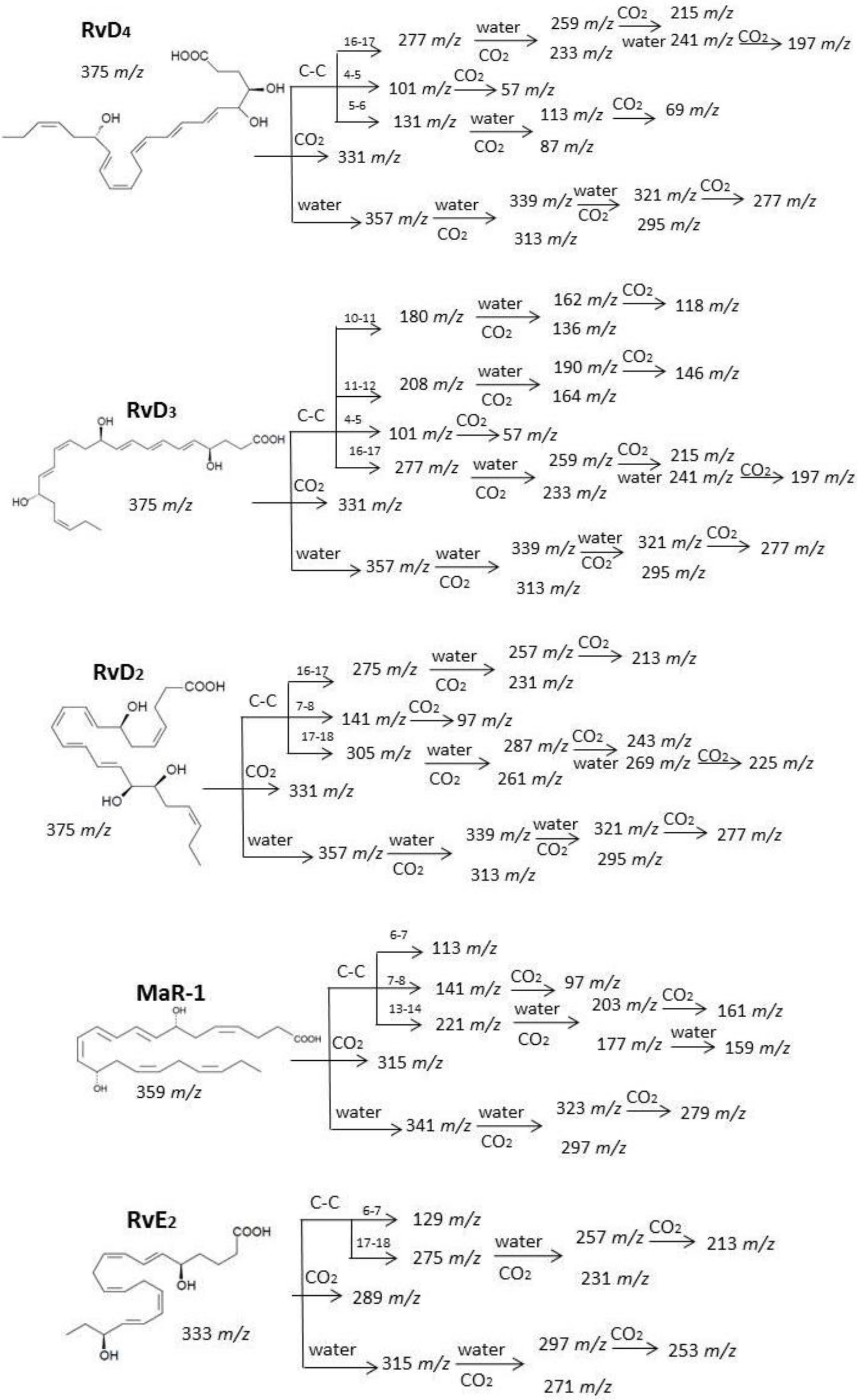

Supplementary Materials S5: Fragmentation pattern suggested for LxB4 (product ion spectrum at *m/z* 351 [33]), and several DiHETEs and DiHETrEs (product ion spectrum at *m/z* 335 and *m/z* 337 obtained from *LIPID MAPS* at CID 30V). MS/MS spectrum was not found for LxA5, 8,15-DiHETE, 14,15-DiHETE, and 17,18-DiHETE; thus, possible fragments for identification were suggested based on lipid structure.

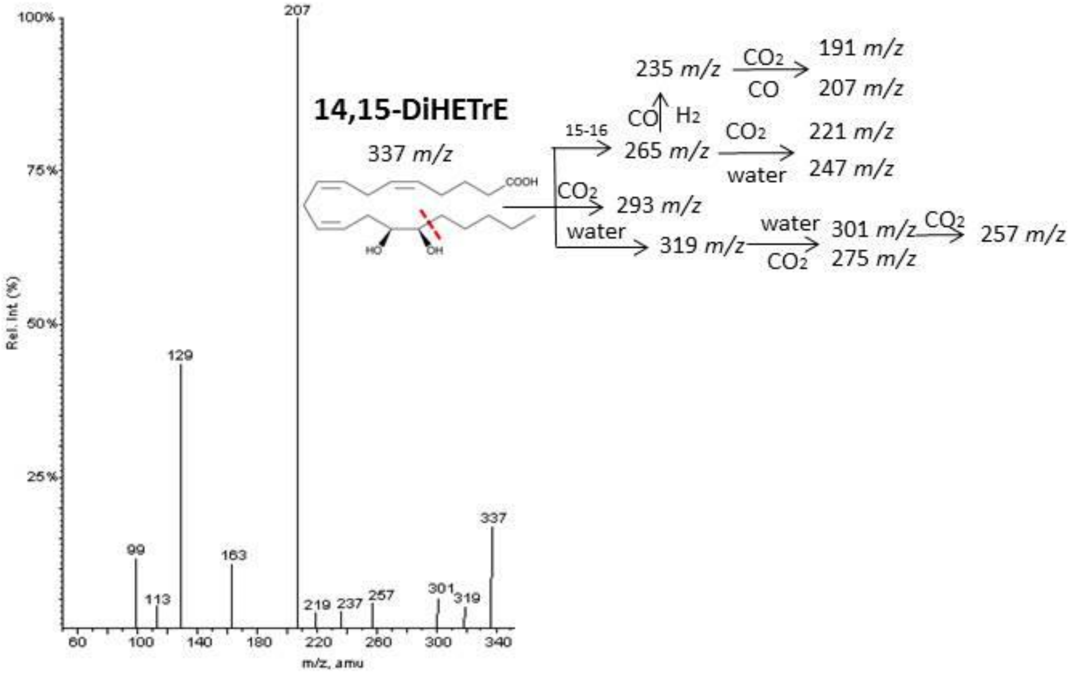

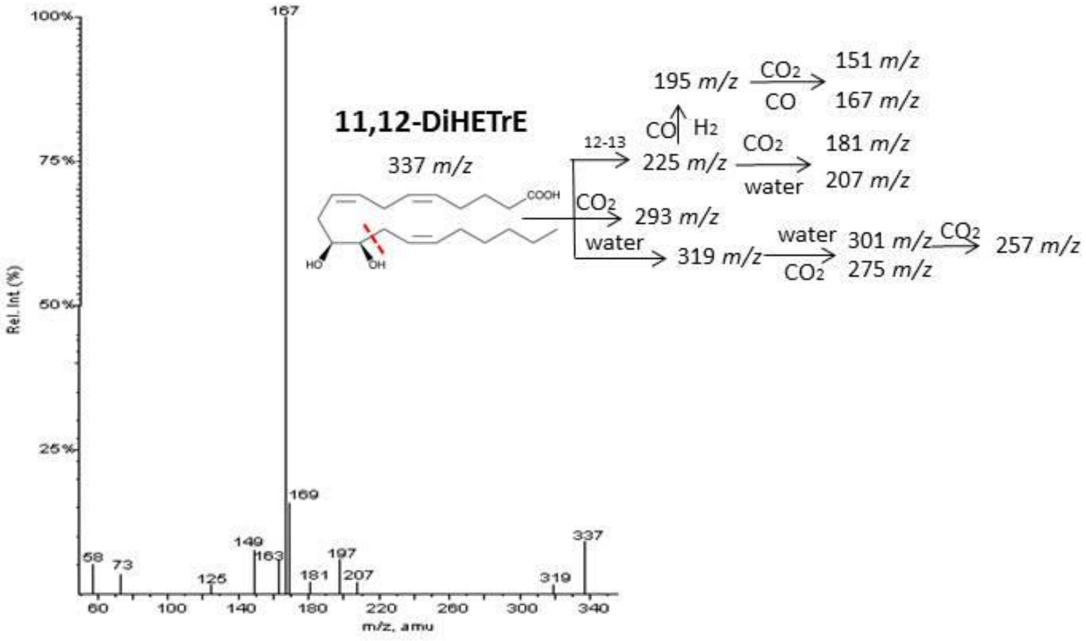

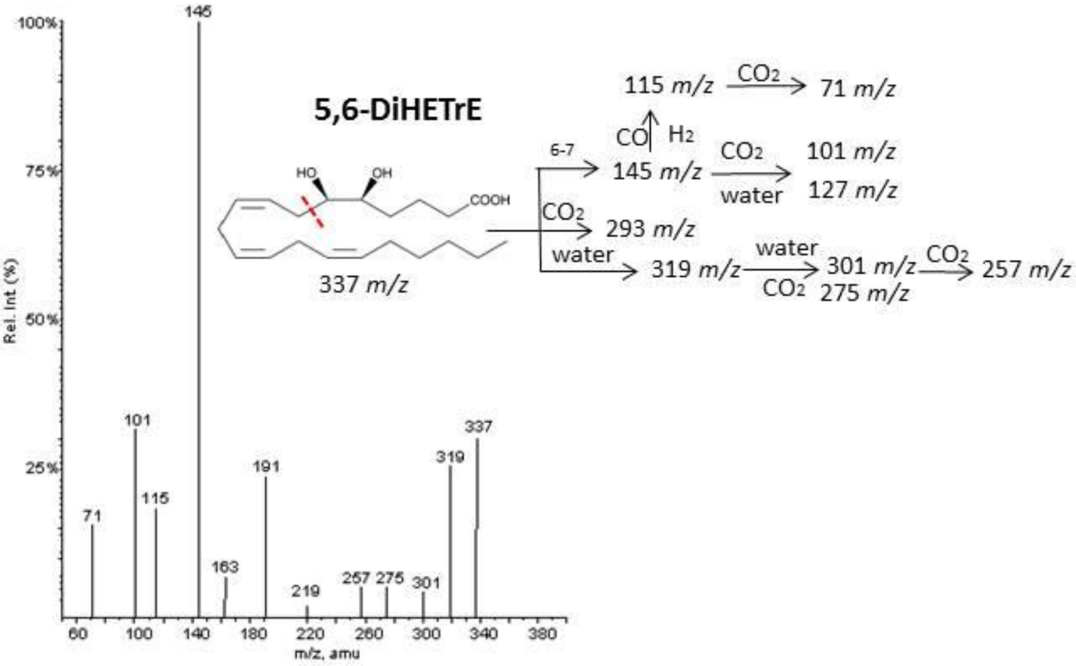
5,6-DiHETrE
337 m/z
115 m/z
CO2
71 m/z
CO
H2
6-7
145 m/z
101 m/z
water
127 m/z
293 m/z
319 m/z
301 m/z
257 m/z
275 m/z
Rel. Int (%)
m/z, amu

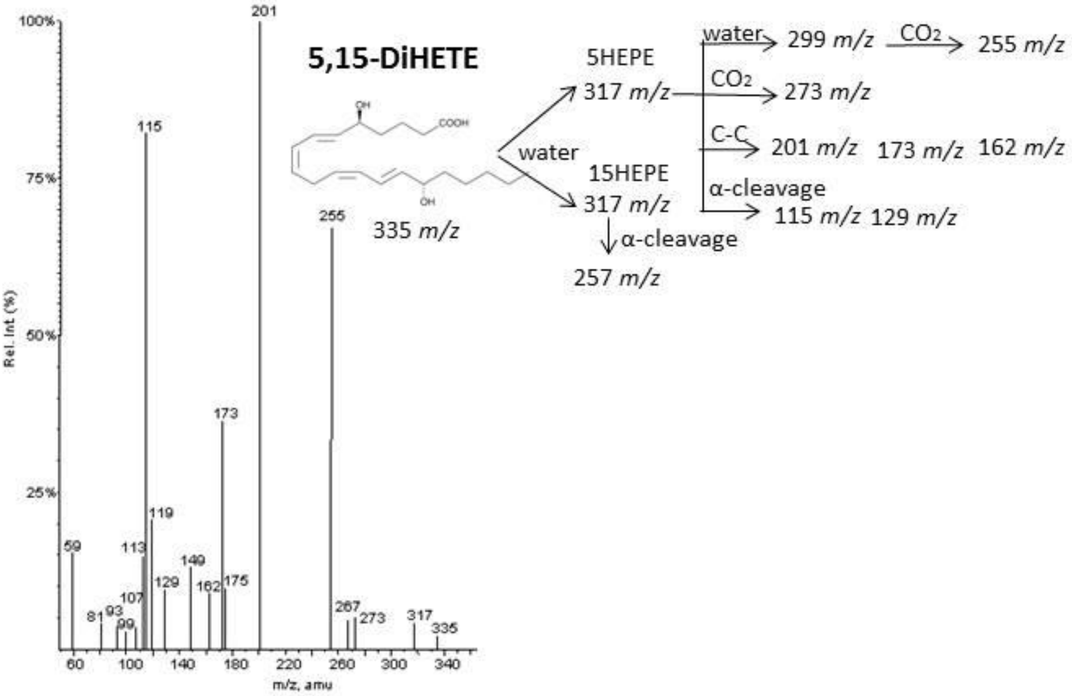
5,15-DiHETE
335 m/z
water
5HEPE
317 m/z
15HEPE
299 m/z
255 m/z
CO2
273 m/z
C-C
201 m/z
173 m/z
162 m/z
α-cleavage
115 m/z
129 m/z
257 m/z
Rel. Int (%)
m/z, amu

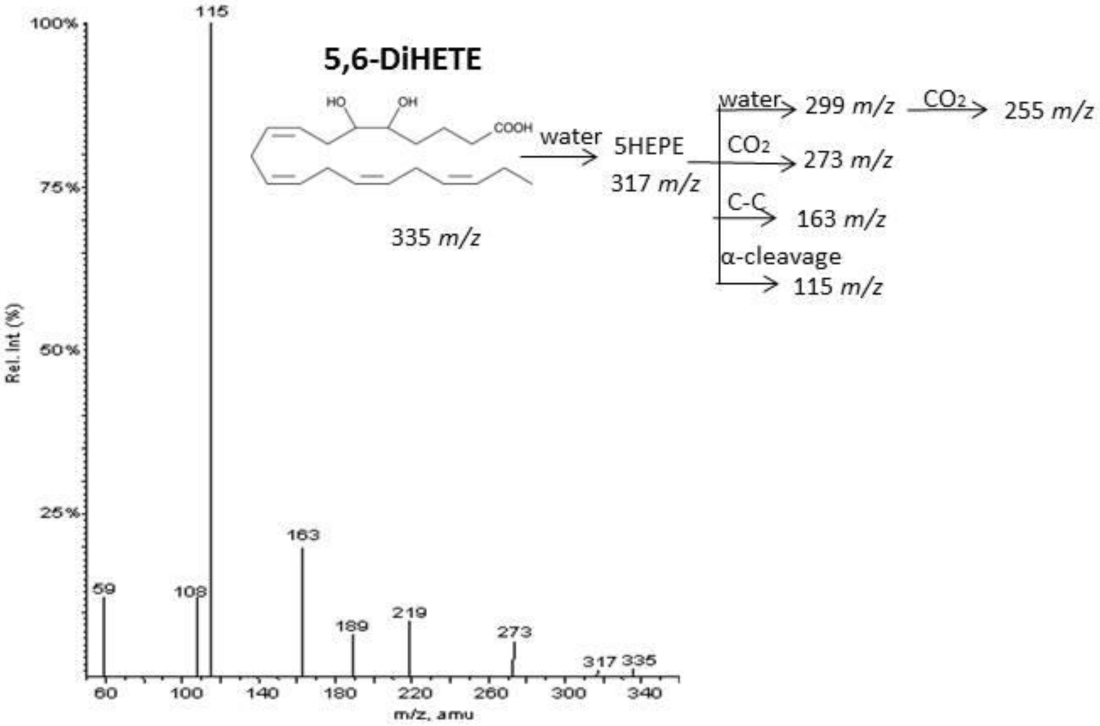
5,6-DiHETE
335 m/z
water
5HEPE
317 m/z
water
299 m/z
CO2
255 m/z
CO2
273 m/z
C-C
163 m/z
α-cleavage
115 m/z
Rel. Int (%)
m/z, amu

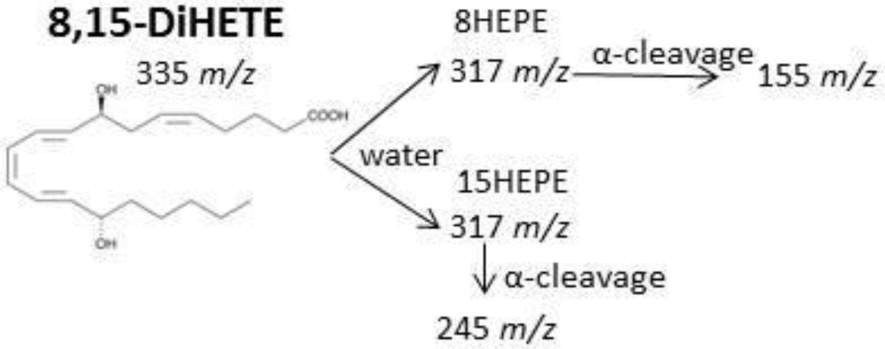
8,15-DiHETE
335 m/z
water
8HEPE
317 m/z
α-cleavage
155 m/z
15HEPE
317 m/z
α-cleavage
245 m/z

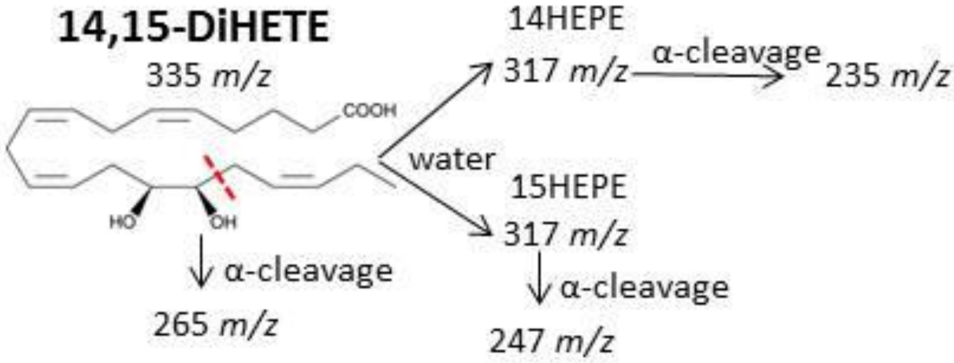
14,15-DiHETE
335 m/z
α-cleavage
265 m/z
water
14HEPE
317 m/z
α-cleavage
235 m/z
15HEPE
317 m/z
α-cleavage
247 m/z

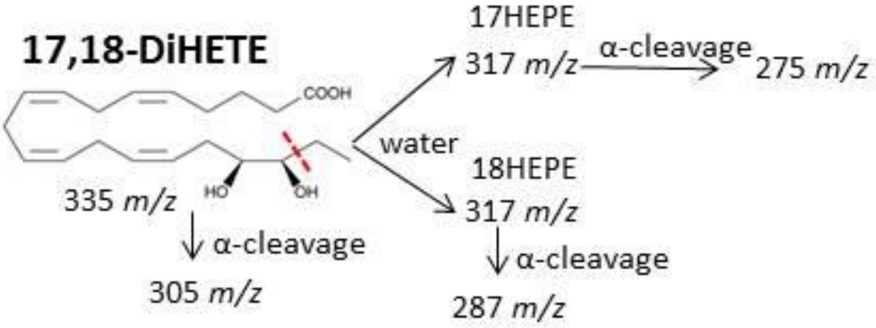
17,18-DiHETE
COOH
HO
OH
335 m/z
α-cleavage
305 m/z
water
17HEPE
317 m/z
α-cleavage
275 m/z
18HEPE
317 m/z
α-cleavage
287 m/z

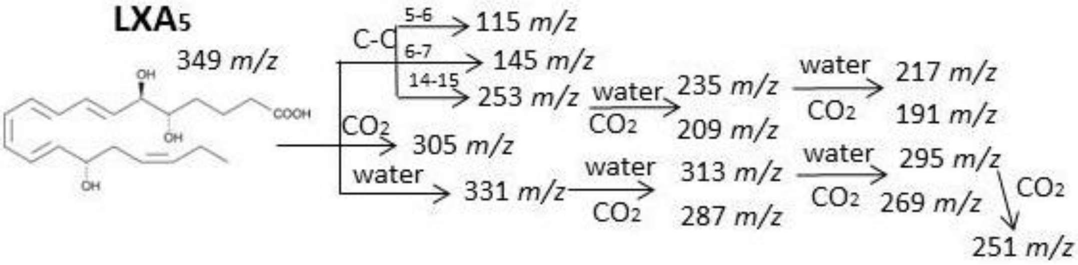
LXA5
349 m/z
OH
COOH
OH
OH
C-C
5-6
115 m/z
6-7
145 m/z
14-15
253 m/z
water
CO2
235 m/z
209 m/z
water
217 m/z
CO2
191 m/z
CO2
305 m/z
water
331 m/z
water
CO2
313 m/z
287 m/z
water
295 m/z
CO2
269 m/z
CO2
251 m/z

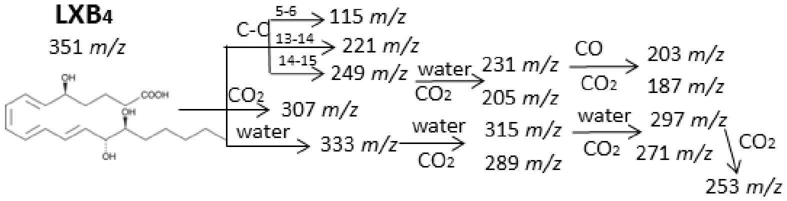
LXB4
351 m/z
OH
COOH
OH
OH
C-C
5-6
115 m/z
13-14
221 m/z
14-15
249 m/z
water
CO2
231 m/z
205 m/z
CO
203 m/z
CO2
187 m/z
CO2
307 m/z
water
333 m/z
water
CO2
315 m/z
289 m/z
water
297 m/z
CO2
271 m/z
CO2
253 m/z

Supplementary Materials S6: Fragmentation pattern suggested for EpETrE isomers and LTA_4. Product ion spectrum at *m/z* 319 were obtained from database of *LIPID MAPS* at CID 30V, and *m/z* 317 was obtained from Murphy et al [33].

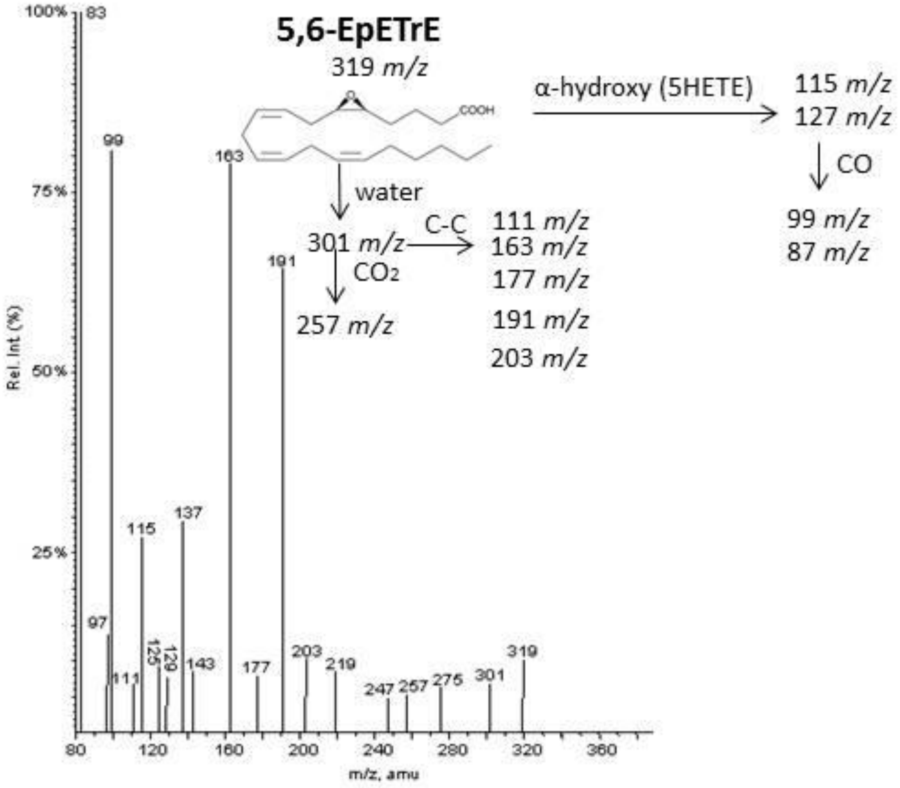

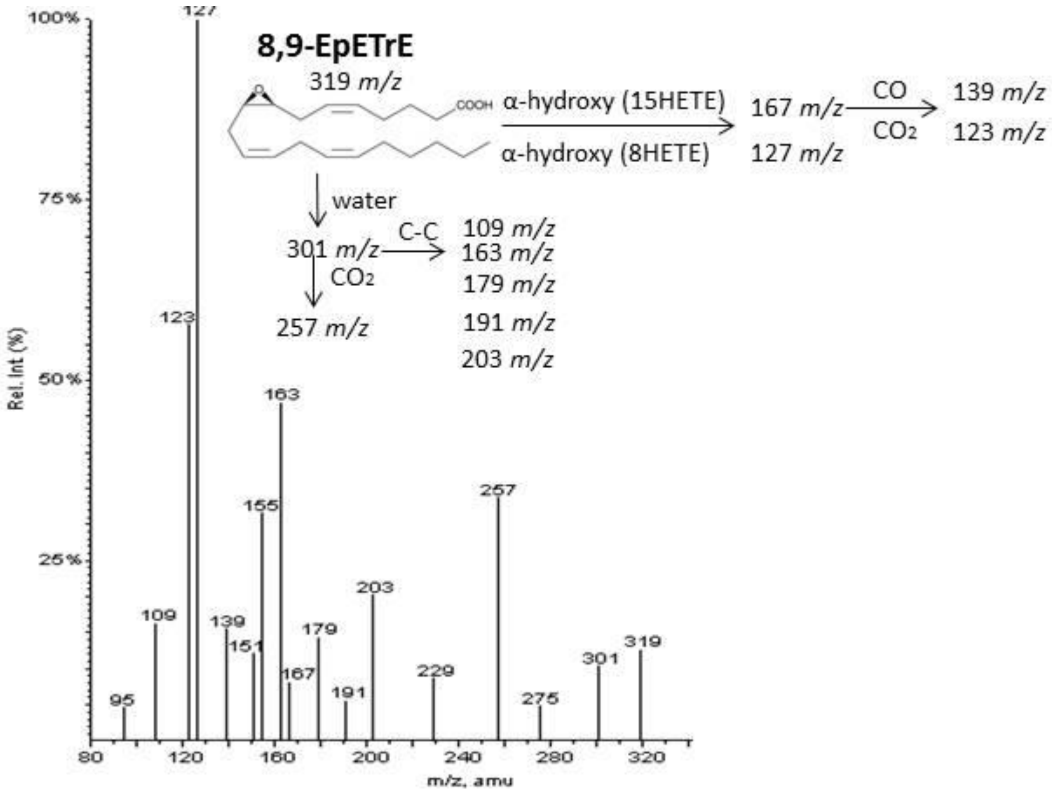

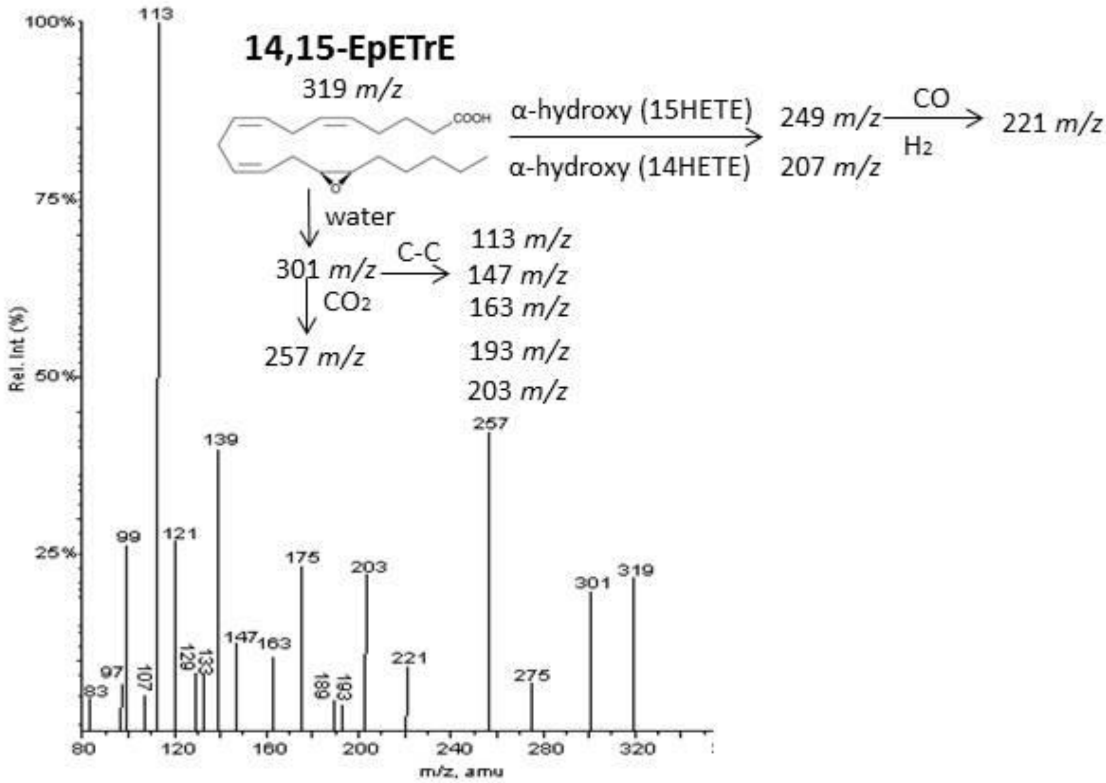
14,15-EpETrE
319 m/z
COOH
α-hydroxy (15HETE)
249 m/z
CO
H2
221 m/z
α-hydroxy (14HETE)
207 m/z
water
301 m/z
C-C
113 m/z
147 m/z
163 m/z
193 m/z
203 m/z
CO2
257 m/z
100%
75%
50%
25%
Rel. Int (%)
113
139
99
121
175
203
257
301
319
147
163
221
275
83
97
107
129
133
189
193
80
120
160
200
240
280
320
m/z, amu

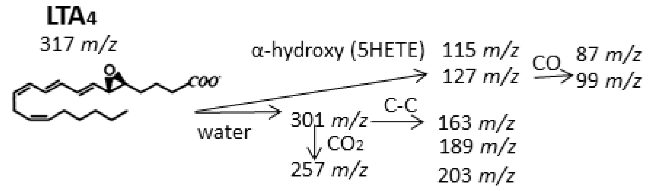
LTA4
317 m/z
COO-
α-hydroxy (5HETE)
115 m/z
127 m/z
CO
87 m/z
99 m/z
water
301 m/z
C-C
163 m/z
189 m/z
203 m/z
CO2
257 m/z

Supplementary Materials S7: Fragmentation pattern suggested for several keto-PGs. Product ion spectrum at *m/z* 349, *m/z* 351, *m/z* 353, *m/z* 367, and *m/z* 369 were obtained from database of *LIPID MAPS* at CID 30V.

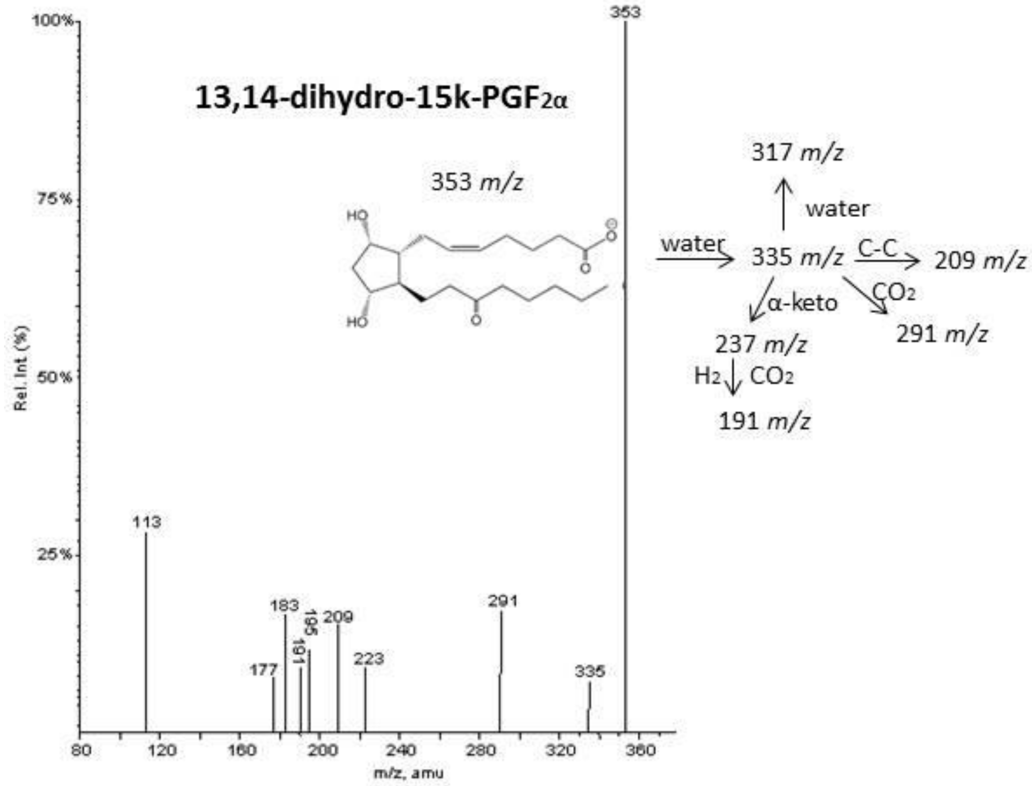

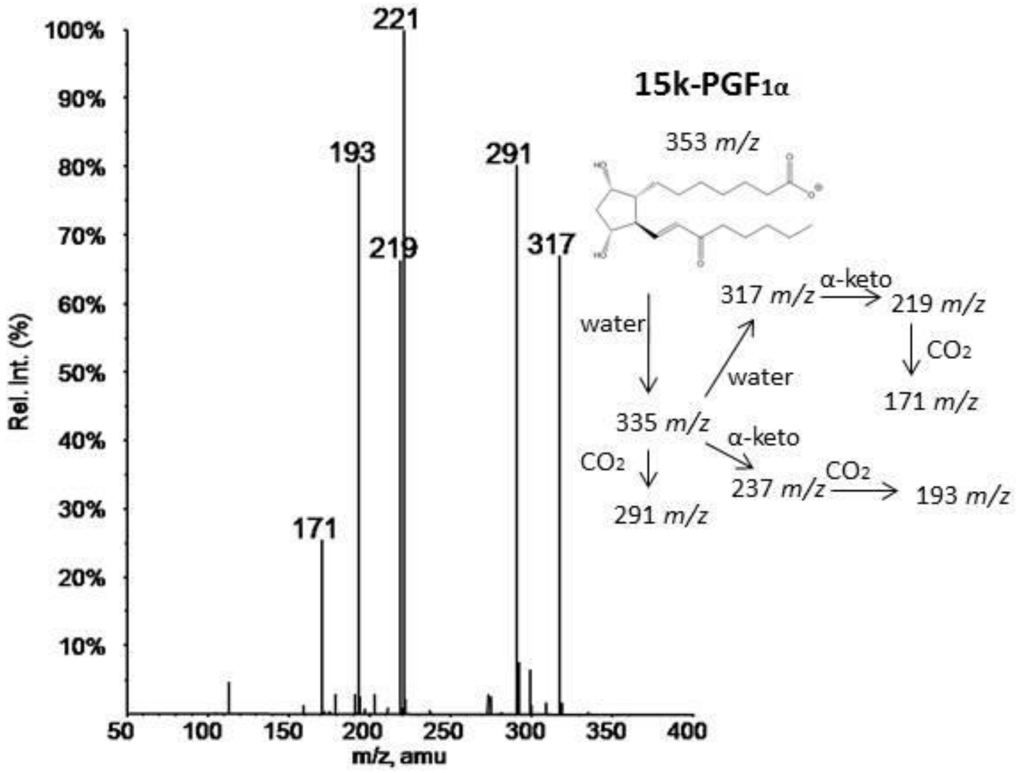

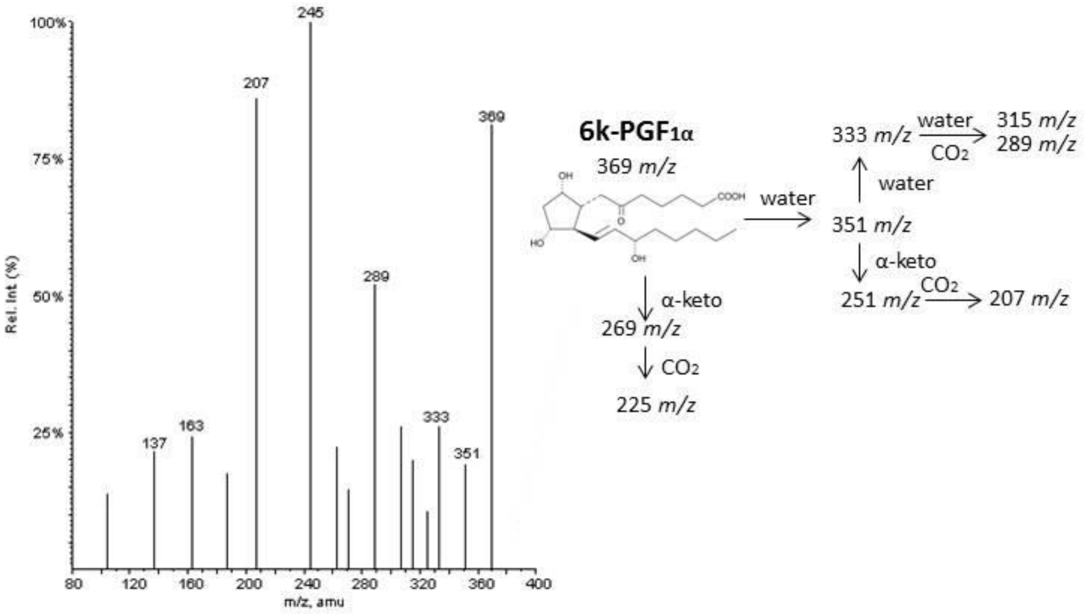
6k-PGF1α
369 m/z
water
351 m/z
water
333 m/z
water
315 m/z
CO2
289 m/z
α-keto
251 m/z
CO2
207 m/z
α-keto
269 m/z
CO2
225 m/z
Rel. Int (%)
m/z, amu

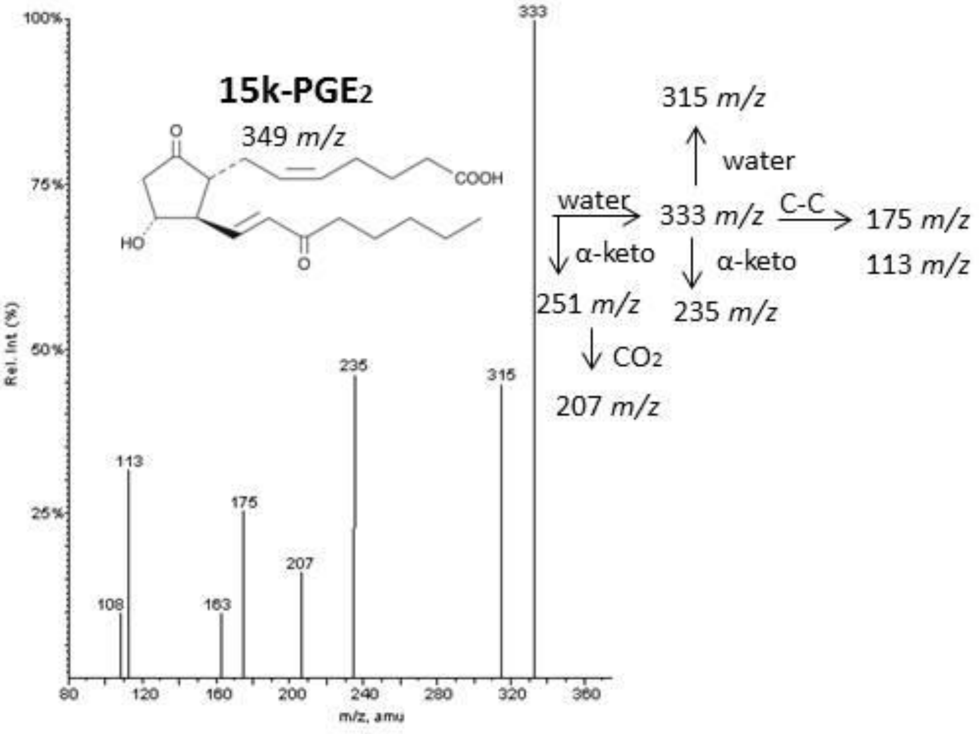
15k-PGE2
349 m/z
water
333 m/z
water
315 m/z
C-C
175 m/z
113 m/z
α-keto
235 m/z
α-keto
251 m/z
CO2
207 m/z
Rel. Int (%)
m/z, amu

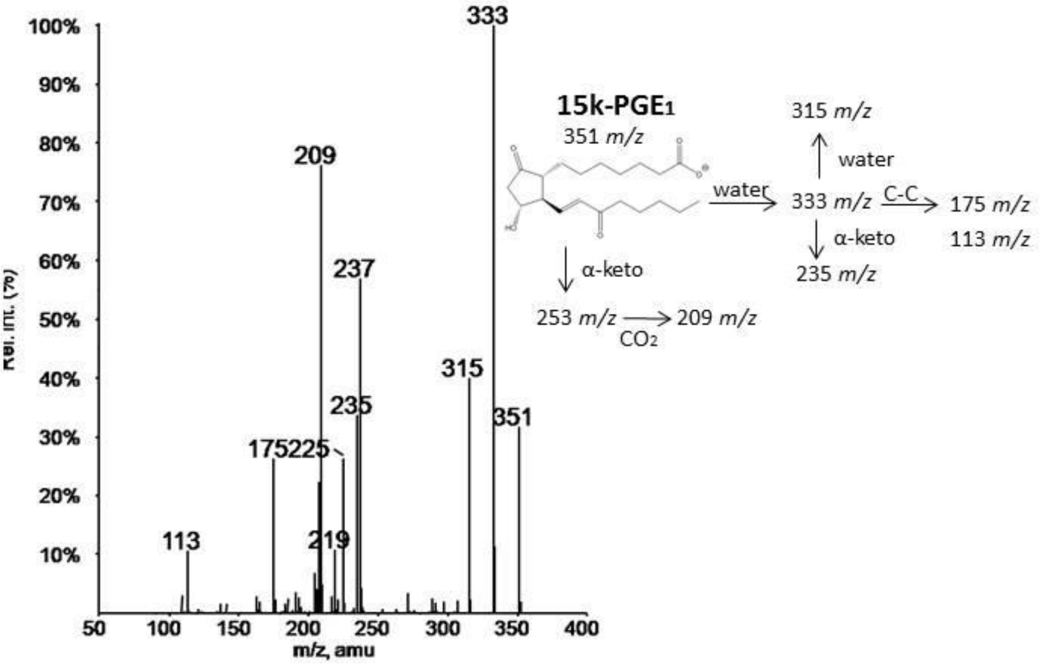
15k-PGE1
351 m/z
water
333 m/z
C-C
175 m/z
113 m/z
315 m/z
α-keto
235 m/z
253 m/z
209 m/z
CO2
Rel. Int. (%)
m/z, amu

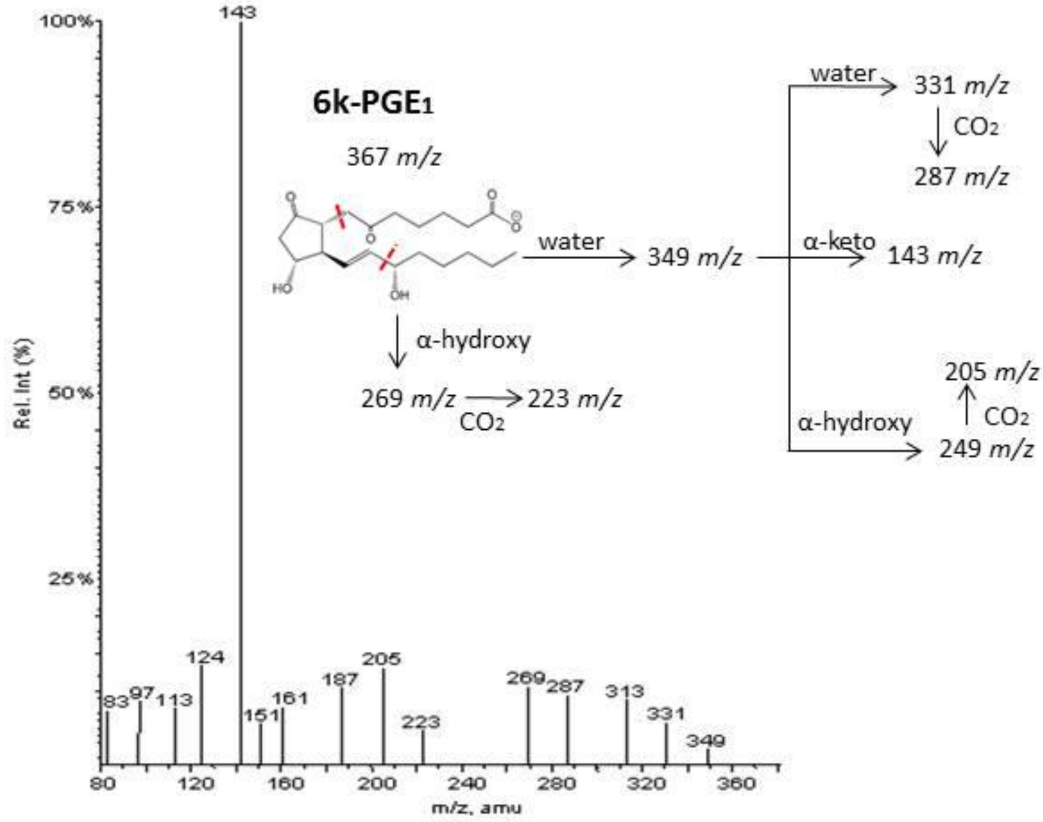
6k-PGE1
367 m/z
water
349 m/z
α-keto
143 m/z
331 m/z
CO2
287 m/z
α-hydroxy
269 m/z
223 m/z
205 m/z
249 m/z
Rel. Int (%)
m/z, amu

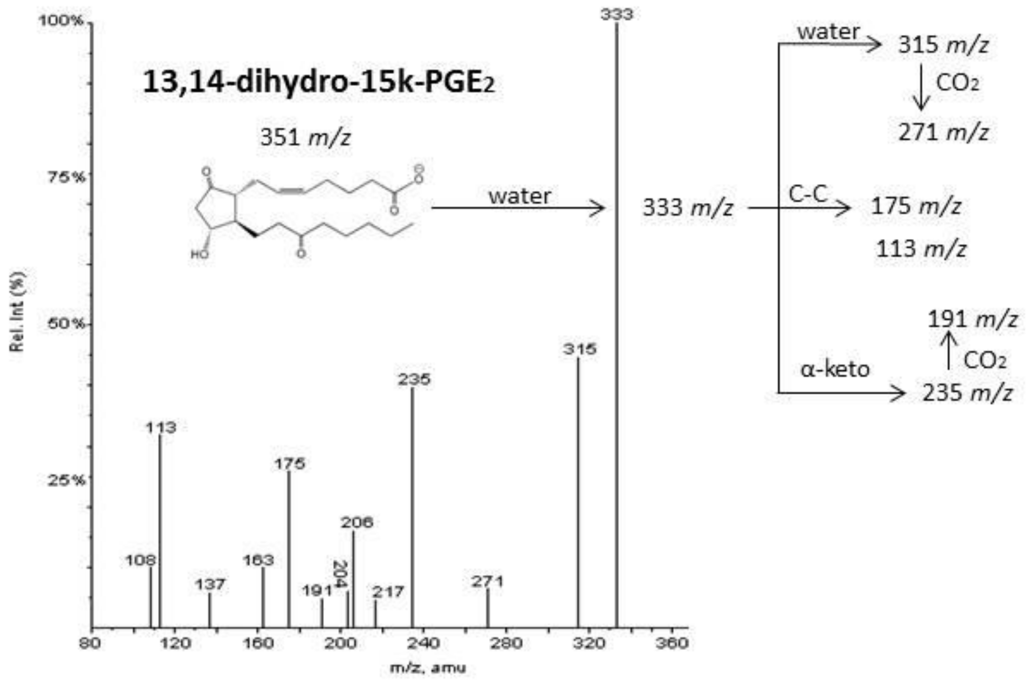
13,14-dihydro-15k-PGE2
351 m/z
water
333 m/z
water
315 m/z
CO2
271 m/z
C-C
175 m/z
113 m/z
191 m/z
CO2
α-keto
235 m/z
Rel. Int (%)
m/z, amu

Supplementary Materials S8: Fragmentation pattern suggested for TXB_2 and TXA_2. The product ion spectrum at and *m/z* 369 was obtained from *LIPID MAPS* at CID 30V. Characteristic MS/MS transitions were suggested for TXA_2 based on lipid structure.

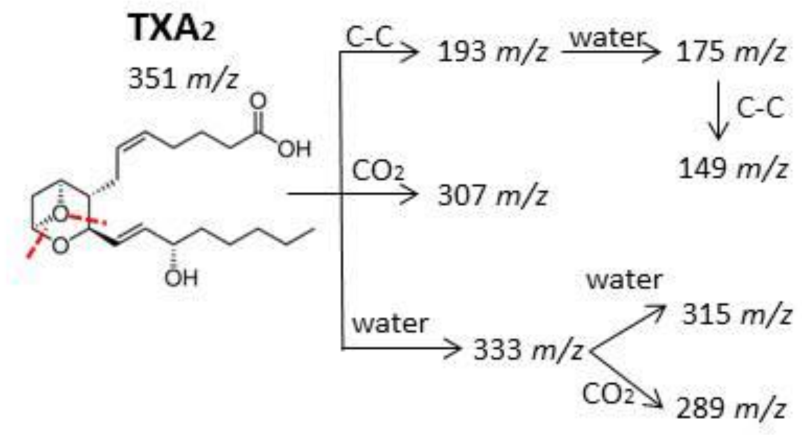

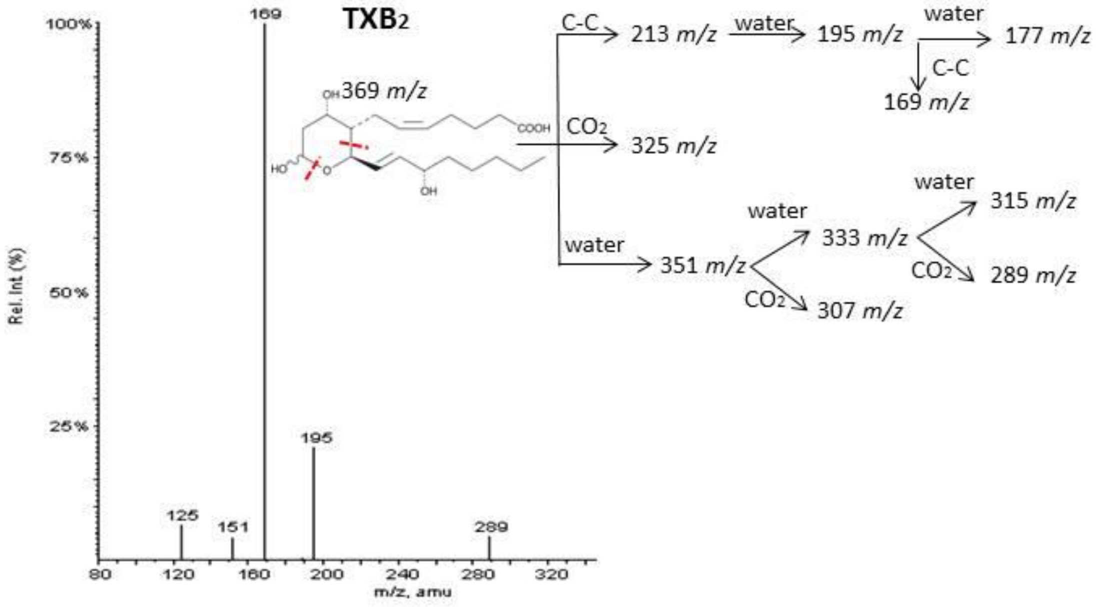

Supplement: Supplementary file 1 [file molecules-24-02276-s001.pdf]
